# The Origin of Internal Genes Contributes to the Replication and Transmission Fitness of H7N9 Avian Influenza Virus

**DOI:** 10.1128/jvi.01290-22

**Published:** 2022-11-07

**Authors:** Joe James, Sushant Bhat, Sarah K. Walsh, Thusitha K. Karunarathna, Jean-Remy Sadeyen, Pengxiang Chang, Joshua E. Sealy, Sahar Mahmood, Benjamin C. Mollett, Marek J. Slomka, Sharon M. Brookes, Munir Iqbal

**Affiliations:** a Animal and Plant Health Agency, Weybridge, United Kingdom; b Avian Influenza Group, The Pirbright Institute, Woking, United Kingdom; Cornell University

**Keywords:** H7N9, H9N2, avian influenza, chicken coinfection, internal genes, poultry, reassortment, transmission

## Abstract

H9N2 avian influenza viruses (AIVs) have donated internal gene segments during the emergence of zoonotic AIVs, including H7N9. We used reverse genetics to generate A/Anhui/1/13 (H7N9) and three reassortant viruses (2:6 H7N9) which contained the hemagglutinin and neuraminidase from Anhui/13 (H7N9) and the six internal gene segments from H9N2 AIVs belonging to (i) G1 subgroup 2, (ii) G1 subgroup 3, or (iii) BJ94 lineages, enzootic in different regions throughout Asia. Infection of chickens with the 2:6 H7N9 containing G1-like H9N2 internal genes conferred attenuation *in vivo*, with reduced shedding and transmission to contact chickens. However, possession of BJ94-like H9N2 internal genes resulted in more rapid transmission and significantly elevated cloacal shedding compared to the parental Anhui/13 H7N9. *In vitro* analysis showed that the 2:6 H7N9 with BJ94-like internal genes had significantly increased replication compared to the Anhui/13 H7N9 in chicken cells. *In vivo* coinfection experiments followed, where chickens were coinfected with pairs of Anhui/13 H7N9 and a 2:6 H7N9 reassortant. During ensuing transmission events, the Anhui/13 H7N9 virus outcompeted 2:6 H7N9 AIVs with internal gene segments of BJ94-like or G1-like H9N2 viruses. Coinfection did lead to the emergence of novel reassortant genotypes that were transmitted to contact chickens. Some of the reassortant viruses had a greater replication in chicken and human cells compared to the progenitors. We demonstrated that the internal gene cassette determines the transmission fitness of H7N9 viruses in chickens, and the reassortment events can generate novel H7N9 genotypes with increased virulence in chickens and enhanced zoonotic potential.

**IMPORTANCE** H9N2 avian influenza viruses (AIVs) are enzootic in poultry in different geographical regions. The internal genes of these viruses can be exchanged with other zoonotic AIVs, most notably the A/Anhui/1/2013-lineage H7N9, which can give rise to new virus genotypes with increased veterinary, economic and public health threats to both poultry and humans. We investigated the propensity of the internal genes of H9N2 viruses (G1 or BJ94) in the generation of novel reassortant H7N9 AIVs. We observed that the internal genes of H7N9 which were derivative of BJ94-like H9N2 virus have a fitness advantage compared to those from the G1-like H9N2 viruses for efficient transmission among chickens. We also observed the generation of novel reassortant viruses during chicken transmission which infected and replicated efficiently in human cells. Therefore, such emergent reassortant genotypes may pose an elevated zoonotic threat.

## INTRODUCTION

Among the many diverse avian influenza viruses (AIVs), the H9N2 subtype of AIVs became prominent by the turn of the century, when they had become enzootic in poultry in many parts of Asia (1). H9N2 AIVs have continued to evolve in the field, by both genetic drift and reassortment, whereby the latter frequently shifts the predominant genotypes observed in poultry, with fitter viruses outcompeting existing strains and emerging as novel dominant strains (2, 3). H9N2 viruses can be classified phylogenetically by the hemagglutinin (HA) gene, which includes three stable lineages, two of which, namely, G1 and BJ94 (sometimes referred to as Y280), are of Asian origin; the former is essentially restricted to terrestrial poultry but has spread to the Middle East and into Africa (4–6). The third Eurasian grouping, Y439, is part of a broader global lineage which also includes H9N2 AIVs from North American wild birds and European isolates (7, 8). Although all H9N2 AIVs are low-pathogenicity AIVs (LPAIVs), they can cause mortality in poultry, particularly when associated with secondary microbial infections (9, 10). In addition, H9N2 viruses continue to be responsible for mild zoonotic infections which have been recorded mainly in East Asia since 1998, but with no evidence thus far for any onward efficient transmission among humans (11).

Within the BJ94 lineage, genotype 57 (G57) H9N2 viruses were first identified in China in 2007 and had become the predominant genotype in poultry due to their enhanced *in vivo* fitness (12). Subsequent reassortment of G57 genotype H9N2 viruses with other circulating subtypes has resulted in the generation of several zoonotic AIVs, including H5N1 (1997 to present), H7N9 (2013 to present), H10N8 (2014), and H5N6 (2015) (13–16). Some of these novel reassortants contained internal genes from the H9N2 G57 genotype and from several H5Nx highly pathogenic avian influenza viruses (HPAIVs) of the “goose/Guangdong” lineage (17–19). Importantly, it has been previously demonstrated that the internal gene segments of G57 greatly contribute to the pathogenicity of these viruses in mammals, further emphasizing their significance as a novel zoonotic threat, whereby their pandemic potential cannot be ignored (13, 14, 17, 18, 20–24).

Following the epizootic emergence of A/Anhui/1/2013-lineage H7N9 during 2013 (prototype virus strain A/Anhui/1/2013 [H7N9]) through an earlier reassortment involving a wild bird H7 AIV and a G57 H9N2, H7N9 incurred extensively into chickens in China as an LPAIV with an absence of overt clinical signs in chickens (15). This H7N9 virus has caused over 1,500 human infections, with a 38% case fatality rate (16), and prompted concerns that it may threaten to evolve into the next human influenza pandemic (25, 26). H7N9 has continued to circulate in poultry and diversified into multiple lineages, in part by dynamically reassorting with other viruses in China, particularly H9N2 viruses (27, 28). Widespread H7 AIV vaccination in poultry in China has resulted in a marked reduction in cases; however, H7N9 continues to be detected in poultry (16). There is an ever-present concern that geographical expansion of H7N9 could facilitate reassortment with a more diverse range of AIVs, leading to the generation of novel AIVs which are more transmissible or pathogenic in poultry and have a higher propensity for zoonotic transmission to humans (29). Indeed, H9N9 and H7N2 subtypes have already been seen to arise as a result of natural reassortment in the field (30–33) or after experimental coinfection between H7N9 and H9N2 (34), with the progeny having been shown to have increased virulence both experimentally and naturally (34, 35). We have previously demonstrated how a novel H9N9 reassortant virus emerged as the dominant genotype following *in vivo* coinfection between A/Anhui/1/2013-lineage H7N9 and G1-like H9N2 viruses (34). This H9N9 reassortant virus transmitted successfully to naive chickens and efficiently replicated in ferrets, with evidence of transmission which may have been mediated through aerosols (34). These observations underlined that further reassortment between these H7N9 and H9N2 viruses may result in a significant public health threat, which emphasized the need for further characterization of such novel reassortments to safeguard both the poultry industry and human health.

We selected different H9N2 viruses representative of the dominant lineages which are circulating in countries neighboring China. Using reverse genetics (RG), we rescued viruses consisting of the HA and neuraminidase (NA) genes from the prototype A/Anhui/1/2013-lineage H7N9 (A/Anhui/1/2013, abbreviated to Anhui/13) and the internal gene cassettes from three distinct H9N2 viruses (BJ94-like, G1 subgroup 2, and G1 subgroup 3) isolated from different geographical regions in Asia. We investigated the contribution of the internal genes from H9N2 viruses upon viral fitness of the reassorted H7N9 genotypes in chickens. In order to identify which constellation of H9N2 internal genes may feature in a viable emergent H7N9 reassortant, we also coinfected chickens with mixtures of Anhui/13 and the reassorted H7N9 viruses possessing the different H9N2 internal gene segments. This approach provided elements of competition between the two infecting viruses, along with an experimental setting for the *in vivo* emergence of reassortants. The coinfection experiments prompted characterization of the viable progeny viruses, which included novel genotypes, along with their accompanying consequences for viral fitness, which were assessed *in vitro* using chicken cells. Finally, we investigated the potential zoonotic risk of these novel reassortant viruses by characterizing their replication in human lung epithelial cells.

## RESULTS

### The internal gene cassettes of H9N2 viruses from different lineages confer different replication and transmission properties to 2:6 reassortant H7N9 in chickens.

We rescued the Anhui/13 (H7N9) virus by reverse genetics (RG). To investigate the effect of other H9N2 internal gene cassettes, we generated three reassortant H7N9 viruses by RG; each virus possessed the surface glycoproteins HA and NA from Anhui/13 H7N9 and the remaining six internal gene cassettes from three genetically different H9N2 viruses. These lineages included two “G1-like” viruses from Pakistan (G1 subgroup 2-like) and Bangladesh (G1 subgroup 3-like) and one “BJ94-like” lineage H9N2 virus from Vietnam (see Fig. S1 and Fig. S2 in the supplementary material). These viruses are hereby referred to as 2:6 H7N9 viruses (Pakistan_2:6_, Vietnam_2:6_, and Bangladesh_2:6_) (Fig. S1B) and were compared for their infectivity, transmissibility, and pathogenicity in chickens.

**FIG 1:**
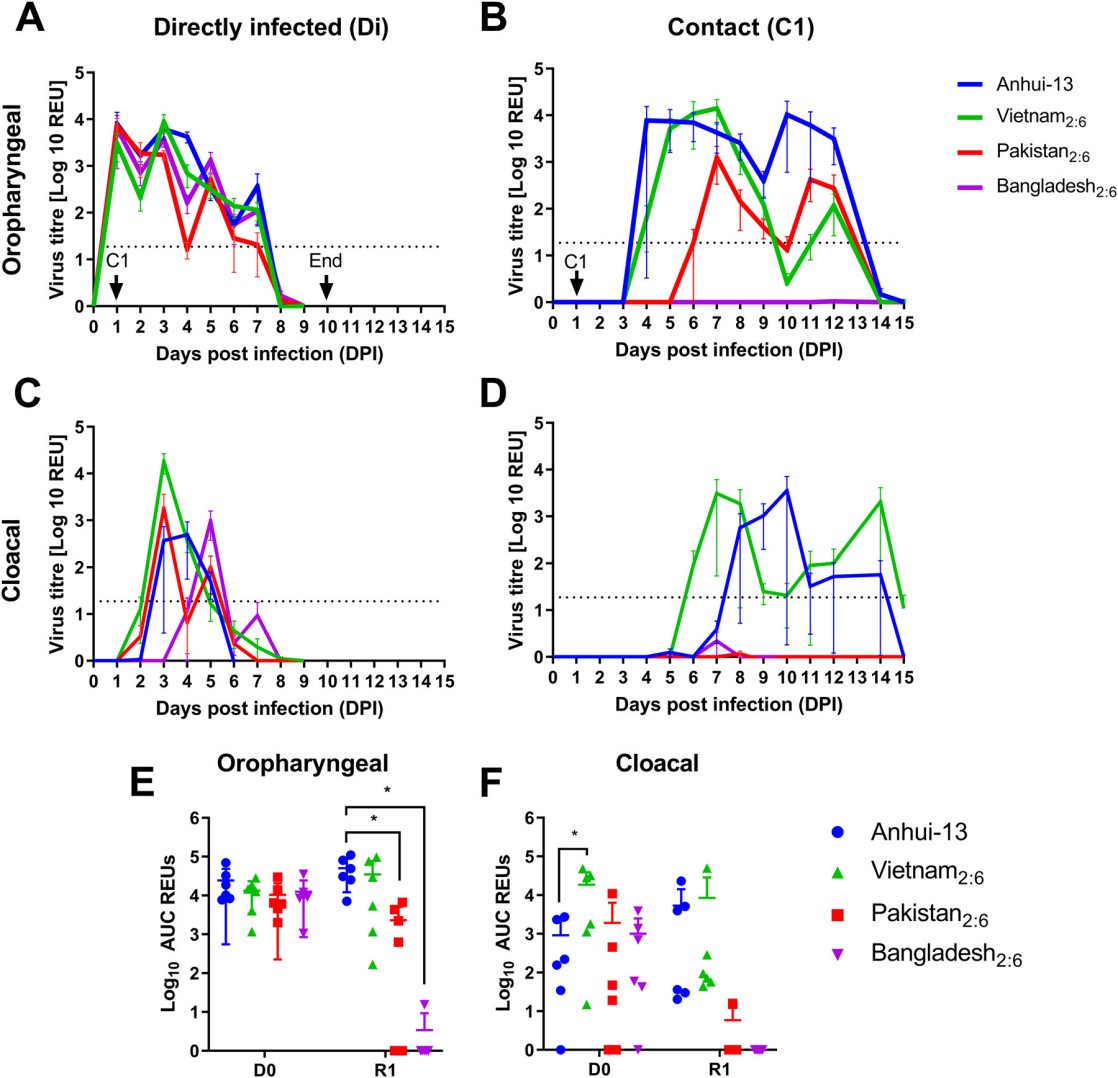
Mean viral shedding and transmission dynamics for Anhui/13 and 2:6 H7N9 viruses possessing different internal gene segments in chickens. (A and C) Mean influenza virus titers (shown as relative equivalence units [REUs] of 50% egg infectious dose [EID_50_]) from swabs taken from chickens directly infected (Di) with 1 × 10^8^ EID_50_ of Anhui (blue) or 2:6 H7N9 viruses possessing the internal gene segments from either Vietnam (green), Pakistan (red), or Bangladesh (purple) H9N2 viruses. (B and D) Mean REUs from contact chickens (C1) placed in contact with infected Di groups at 1 dpi. Swabs were taken from the oropharyngeal (A and B) or cloacal (C and D) cavities of directly infected or contact chickens. Each colored line represents mean shedding ± standard deviation (SD). Oropharyngeal shedding profiles from individual birds are shown in Fig. S3. REUs and positive cutoffs are described in Materials and Methods; negative detections were given a value of 0 REUs. Individual shedding is shown in Fig. S3. (E and F) Area under the curve (AUC) analysis for oropharyngeal (E) and cloacal (F) shedding. Each point represents the AUC for an individual chicken; lines represent the mean ± SD. The asterisks (*) indicate a *P* value of <0.05 from a one-way analysis of variance (ANOVA) with multiple comparisons.

For the single-virus infections, chickens directly infected (Di) with Anhui/13 registered mean positive shedding from the oropharyngeal cavity at 1 day postinfection (dpi) with peak viral RNA titers between 1 and 4 dpi, which ceased by 8 dpi (Fig. 1A and Fig. S3). The Vietnam_2:6_, Pakistan_2:6_, and Bangladesh_2:6_ viruses all caused robust infection in Di chickens (Fig. 1A), producing total oropharyngeal shedding profiles similar to those of Anhui/13 (Fig. 1E). For all viruses, cloacal shedding was more sporadic than oropharyngeal shedding, observed between 2 and 7 dpi (Fig. 1C). However, cloacal shedding in the Di Vietnam_2:6_ chickens was significantly greater than that of chickens infected with Anhui/13 (Fig. 1F). Chickens from all groups exhibited mild-moderate clinical signs with no significant differences observed among the different groups (data not shown).

Anhui/13 successfully transmitted to 100% of the naive chickens placed in contact (C1; “R” denoting recipient) at 1 dpi, based on viral RNA shedding and seroconversion (Fig. 1B and Fig. S4). Three C1 chickens shed virus from 4 and 5 dpi, with the remaining three shedding from 7 and 8 dpi, after Di shedding was resolving, suggesting that two rounds of transmission may have occurred (Fig. 1B and Fig. S3E). Vietnam_2:6_ also transmitted with 100% efficiency, albeit more rapidly than Anhui/13, where an earlier onset of shedding was apparent (Fig. 1B). The transmission efficiency in the Pakistan_2:6_ group was 66.66% (4 out of 6), with shedding first being observed between 6 and 9 dpi, and 4 out of 6 chickens were seroconverted (Fig. 1B and Fig. S4). No transmission (0 out of 6) occurred in the Bangladesh_2:6_ group based on viral RNA shedding and seroconversion (Fig. 1B and Fig. S4). Only the C1 birds in the Pakistan_2:6_ and Bangladesh_2:6_ virus groups showed a statistically significant decrease in oropharyngeal shedding compared to Anhui/13 (Fig. 1E). Among the C1 birds, only the Anhui/13 and Vietnam_2:6_ groups demonstrated sporadic cloacal shedding (Fig. 1D and F), while the other groups did not show any shedding through the cloacal route. The Vietnam_2:6._ group showed significantly increased cloacal shedding compared to the Anhui/13 group.

Taken together, these results indicated that the internal gene cassettes from the Vietnam H9N2 virus bestow increased cloacal shedding together with more rapid transmission dynamics, along with the same high transmission efficiency observed in the Anhui/13 group. In contrast, the internal gene cassettes from the Pakistan_2:6_ virus displayed reduced transmission efficiency compared to Anhui/13. More strikingly, the Bangladesh H9N2 viruses resulted in reduced viral shedding and no transmission to naive chickens.

### *In vivo* competition experiments in chickens coinfected with a mixture of Anhui/13 H7N9 and the 2:6 reassorted H7N9 viruses.

**FIG 2:**
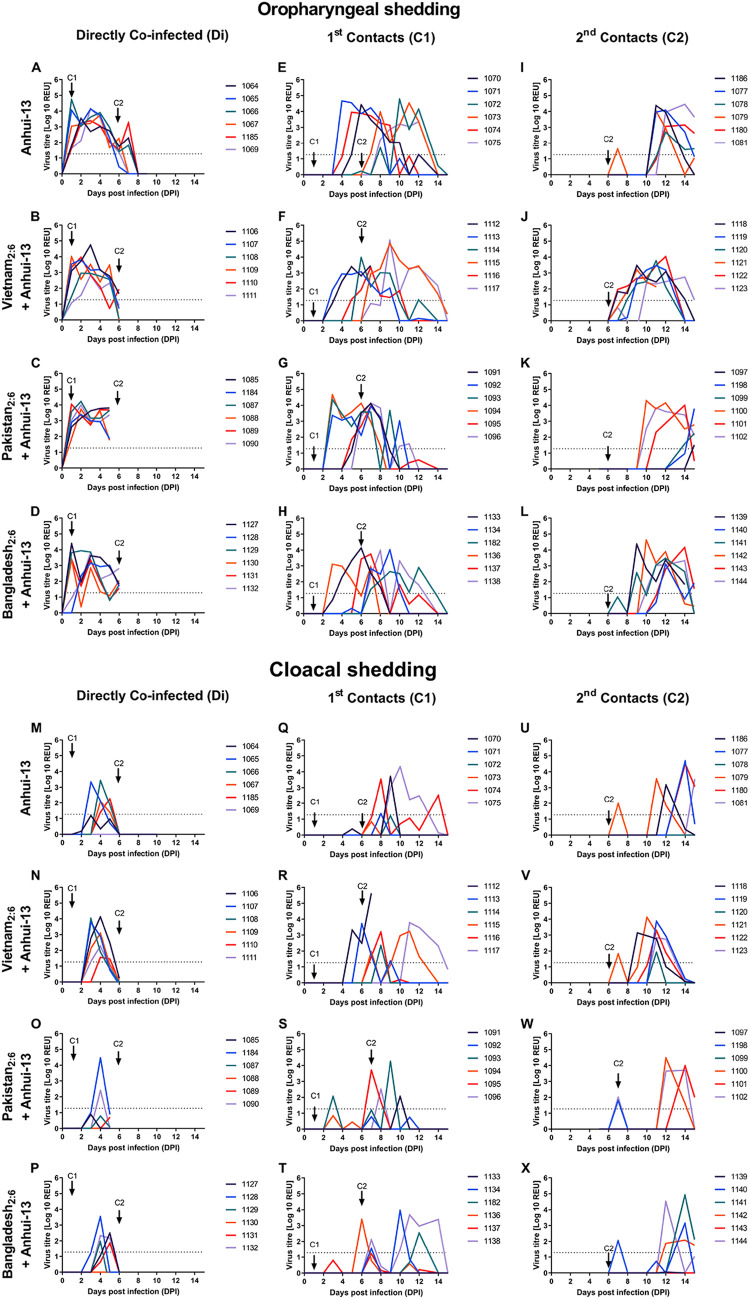
Viral shedding from individual chickens coinfected with both Anhui/13 and one of the three 2:6 H7N9 viruses possessing different internal gene segments and transmission to contact chickens. (A to X) Influenza virus titers (shown as relative equivalence units [REUs] of 50% egg infectious dose [EID50]) from (A to L) oropharyngeal or (M to X) cloacal swabs from chickens infected with 1 × 10^8^ EID_50_ of Anhui (A, E, I, M, Q, and U) or coinfected with 1 × 10^8^ EID_50_ of Anhui and 1 × 10^8^ EID_50_ of 2:6 H7N9 viruses possessing the internal gene segments from either Vietnam (B, F, J, N, R, and V), Pakistan (C, G, K, O, S, and W), or Bangladesh (D, H, L, P, T, and X) H9N2 viruses. Shedding titers from chickens placed in contact with infected groups at 1 dpi (1st contacts [C1]) (E to H and Q to T; oropharyngeal and cloacal, respectively) or placed in contact with the 1st contacts (2nd contacts [C2]) (I to L and U to X; oropharyngeal and cloacal, respectively). Each colored line represents shedding from an individual chicken. Arrows indicate the times at which C1 and C2 contact chickens were introduced for successive cohousing periods. REUs and positive cutoffs are described in Materials and Methods.

We next performed a series of *in vivo* coinfection experiments using Anhui/13 H7N9 and each of the 2:6 H7N9 viruses (Pakistan_2:6_, Vietnam_2:6_, or Bangladesh_2:6_) which were administered to Di chickens. Two subsequent rounds of contact chickens (C1 and C2) were introduced in an attempt to establish an onward chain of transmission. Di chickens coinfected with the Anhui/13 and each 2:6 H7N9 virus demonstrated a similar oropharyngeal and cloacal shedding pattern compared to Anhui/13 alone (Fig. 2A to D). The C1 chickens introduced at 1 dpi began shedding from the oropharyngeal cavity from 3 dpi (2 days postcontact [dpc]) (Fig. 2E to H). Based on viral RNA shedding and seroconversion, transmission efficiency to the C1 chickens was 100% in all groups (Fig. 2E to H and Fig. S5). A group of second contacts (C2) were added upon removal of the Di chickens. Again, viral shedding indicated 100% transmission efficiency to the C2 chickens in all groups (Fig. 2I to L). However, not all the C2 chickens seroconverted to H7N9 antigen (Fig. S5), likely because blood sampling was carried out at 14 dpi and some C2 chickens could not seroconvert early. Cloacal shedding was detectable, yet sporadic, for all groups and typically mimicked oropharyngeal shedding, typically commencing at 2 days after onset of oropharyngeal shedding (Fig. 2M to X).

To investigate the constellation of viral gene segments in the C1 and C2 groups following initial Di coinfection, lineage-specific reverse transcriptase quantitative PCRs (RT-qPCRs) were developed for each internal gene segment (PB2, PB1, PA, NP, M, and NS). This approach enabled the specific quantification of each of the segments from Anhui/13 and the six internal gene segments of the three 2:6 H7N9 viruses.

**FIG 3:**
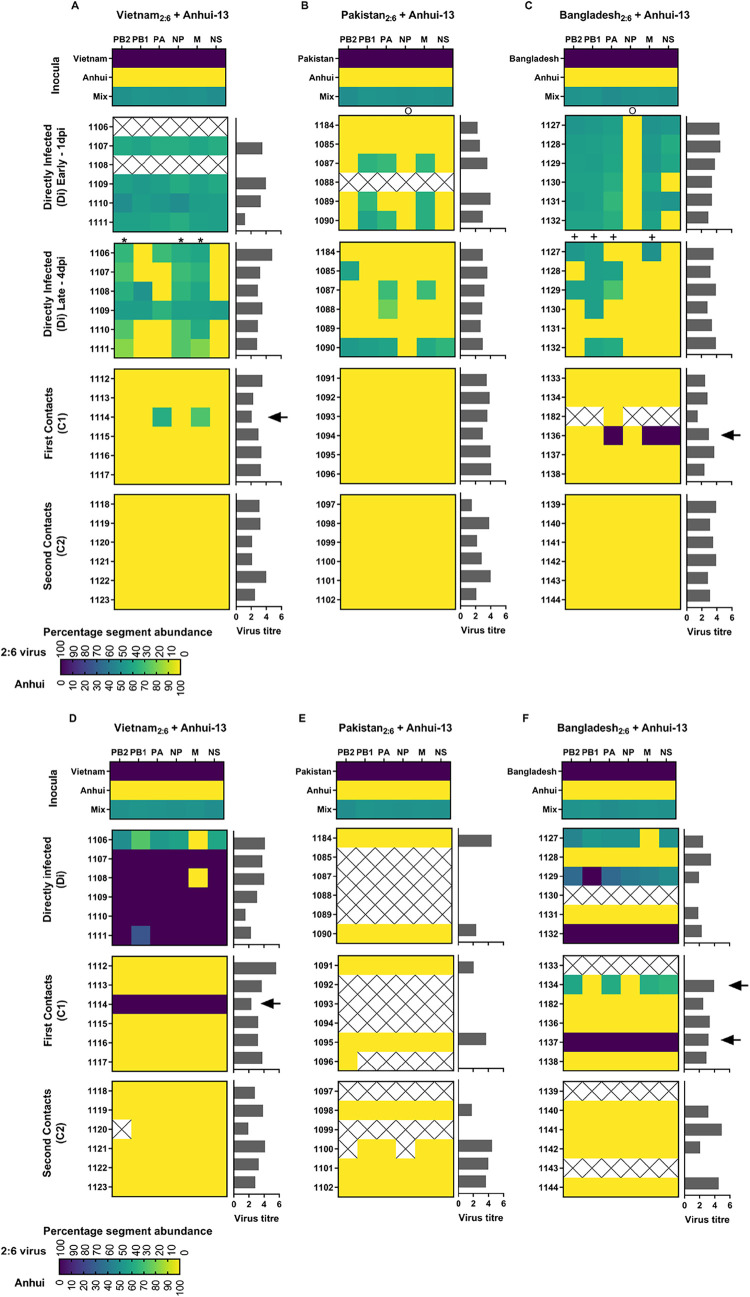
Proportions of distinct internal gene segments among potential progeny viruses from chickens coinfected with Anhui/13 and three 2:6 H7N9 viruses possessing different internal gene segments. Progeny viruses were identified at the directly infected (Di, divided into early [1 dpi] and late [4 dpi] for the oropharyngeal shedding) stages and at the first (C1) or second (C2) contact stage. Swabs from the highest peak viral shedding were analyzed unless stated. (A to F) Heat map colors denote the relative percent abundance of each internal gene segment from viruses in oropharyngeal (A to C) and cloacal (D to F) swabs obtained from the three consecutive transmission stages. The 100% Anhui/13 segment abundance is shown in yellow; 100% 2:6 virus gene abundance is shown in purple; mixed gene abundance is denoted by blended colors. Crosses indicate no gene detection for either virus. Quantified viral shedding titers for each of the analyzed swabs are indicated by the horizontal gray bars (REUs, as determined by the M gene RT-qPCR). Arrows, asterisks, circles, and plus symbols indicate gene segments of interest noted in Results.

The viruses used for the inocula displayed equal (~50%) abundance of Anhui/13 and each 2:6 H7N9 virus gene segment (Fig. 3). In oropharyngeal swabs from Di chickens at 1 dpi, both Anhui/13 and 2:6 H7N9 internal gene segments were identified in all groups, albeit at different relative abundances (Fig. 3). Interestingly, oropharyngeal swabs sampled at 1 dpi from the Di chickens from the Anhui/13 plus Vietnam_2:6_ (Fig. 3A) and Anhui/13 plus Bangladesh_2:6_ (Fig. 3C) groups displayed an increased presence of Vietnam- and Bangladesh-origin internal gene segments, respectively. However, a decline in the frequency of H9N2 internal gene segments was already apparent within these oropharyngeal swabs at the later (4 dpi) Di shedding stage. Indeed, Anhui/13-origin gene segments were thereafter predominantly detected at the C1 stage and by the C2 stage appeared to have successfully out-competed the other H9N2-origin segments, regardless of the mixture administered at the initial Di coinfection (Fig. 3). The restoration of what appeared to be a pure Anhui/13 genotype at the C2 stage following all three initial Di coinfections was observed in both oropharyngeal and cloacal swabs. It was clear that onward *in vivo* transmission in chickens had affected a powerful selection which favored the Anhui/13 internal gene cassette.

### Detection of other potential reassortants at the Di and C1 stages of transmission.

Prior to the exclusive emergence of Anhui/13 at the C2 stage in all three groups, internal segments from all three 2:6 H7N9 viruses were detected during the Di and C1 stages at different frequencies. These observations suggested that several potential reassortant viruses emerged as part of a transient mixed population of progeny viruses (Fig. 3). In the Vietnam_2:6_ plus Anhui/13 coinfected group, mixed PB2, NP, and M gene segments were detected in the oropharyngeal samples at the later Di stage (Fig. 3A, asterisks). At the C1 stage, potential reassortants were identified from chicken 1114, which contained the PA and M segments from Vietnam at a relative percent abundance of 38.5% and 28.5%, respectively, with the remaining six gene segments from Anhui/13 at 100% abundance (Fig. 3A, arrow), potentially indicating two reassortants with different combinations of the PA and M gene segments. Interestingly, the same chicken also shed viral RNA consisting of purely Vietnam internal segments from the cloacal cavity (Fig. 3D).

In the Pakistan_2:6_ plus Anhui/13 coinfected group, potential reassortment was lower than that for the other two groups, where this difference was discernible during oropharyngeal shedding at the early Di stage (compare Fig. 3B with Fig. 3A and C). Pakistan_2:6_ internal segments were not detectable at any of the transmission stages (C1 or C2) from both shedding tracts (Fig. 3B and E). While chicken 1090 showed potential reassortant viruses in all gene segments, except NP, at the later Di stage (4 dpi), three other chickens in this group also showed generation of single- or double-potential gene reassortants in the PB2, PA, or M gene segments. However, either the cloacal samples showed only Anhui/13 H7N9-origin gene segments, or due to low/undetectable virus titers, genotyping could not be performed.

The oropharyngeal samples at the late Di stage in the Bangladesh_2:6_ plus Anhui/13 coinfected group displayed potential reassortant viruses containing PB2, PB1, PA, and M segments originating from Bangladesh_2:6_ with varying abundance (Fig. 3C, plus signs). The cloacal samples from some Di chickens included shedding of internal gene segments of Bangladesh_2:6_ (chicken 1137) or Anhui/13 origin (chicken 1128 and chicken 1131) with 100% abundance (Fig. 3F). The other Di chickens either predominantly shed a mix of internal gene segments from Bangladesh_2:6_, or due to the low virus titer, the genotype could not be identified. The C1-stage oropharyngeal samples revealed one potential reassortant in chicken 1136 which contained the PA, M, and NS gene segments from Bangladesh (with a 100% abundance for all three segments) and all other gene segments from Anhui/13 H7N9 (with 100% abundance) (Fig. 3C, arrow). Cloacal swabs at the C1 stage revealed Bangladesh internal segments in two chickens (1134 and 1137) (Fig. 3F, arrow). Chicken 1134 included the PB2 (59.3%), PA (61.5%), M (62.2%), and NS (65.1%) gene segments of Bangladesh origin, with the remaining gene segments from Anhui/13. Chicken 1137 contained all six internal gene segments from Bangladesh with 100% abundance (Fig. 3F). The potential viral progeny produced in the respiratory and enteric tracts of C1 chickens 1134 and 1137 clearly differed as regards the detected frequency of the various gene segments.

In the Pakistan_2:6_ plus Anhui/13 and Bangladesh_2:6_ plus Anhui/13 coinfected groups, the potential reassortants identified in the oropharyngeal samples of all Di chickens included the NP gene originating from Anhui/13 H7N9, which was maintained throughout subsequent transmission (Fig. 3B and C, circles). The Di cloacal samples from the Bangladesh_2:6_ plus Anhui/13 coinfected group, however, did include shedding of NP from Bangladesh_2:6_, but again, any potential reassortants were outcompeted by viruses containing internal gene segments of Anhui/13 H7N9 origin during the ensuing transmission stages (Fig. 3F). These results showed that NP originating from any other H9N2 virus was rapidly outcompeted by the Anhui/13 NP in both the respiratory and intestinal tracts.

### Plaque-purification of viable reassortant progeny viruses from contact chickens.

To confirm the exact gene constellation of any viable reassortant viruses which successfully transmitted to contact chickens, we performed plaque purification on swabs from C1 chickens which contained detectable 2:6 H7N9 virus gene segments. The selected specimens included oropharyngeal swabs from chickens 1114 and 1136, plus cloacal swabs from chickens 1114, 1134, and 1137 (Fig. 3, arrows). We isolated at least 24 plaques per swab and used the segment-specific RT-qPCRs to determine the genetic constellation of each isolated plaque. The plaque purification findings matched the RT-qPCR genotyping of the corresponding swab specimens and therefore confirmed the viability of bona fide reassortant viruses (Fig. 3 and Table 1). In total, five novel reassortant viruses, with different genetic constellations, were recovered from swabs collected at the C1 stage (H7N9-C1-V29, 30, 31, 32, and 33). The Bangladesh_2:6_ coinfected group produced three new reassortants, one virus as the only population in the oropharyngeal swab from chicken 1136 (genotype H7N9-C1-V29), which contained the Bangladesh-origin PA, M, and NS, and the remaining gene segments from Anhui/13 (Table 1). Variations of this genotype were also shed from the cloacal cavity of chicken 1134, including viruses containing the Bangladesh-origin PB2, PA, M, and NS (genotype H7N9-C1-V33) and PB2, PA, and M (genotype H7N9-C1-V30), with the remaining gene segments from Anhui/13. The Vietnam_2:6_ coinfected group produced two new genotypes from the oropharyngeal cavity of chicken 1114; these viruses contained the Vietnam-origin PA (genotype H7N9-C1-V32) plus PA and M gene segments (genotype H7N9-C1-V31), with the remaining gene segments from Anhui/13 (Table 1). Unaltered Vietnam_2:6_ and Bangladesh_2:6_ (together with unaltered Anhui/13) were also identified by plaque purification, which again reflected the possible viable genotypes indicated earlier (Fig. 3 and Table 1).

**TABLE 1:** The genetic constellation via plaque purification of potential reassortant viruses identified via RT-qPCR from swabs of C1 contact birds

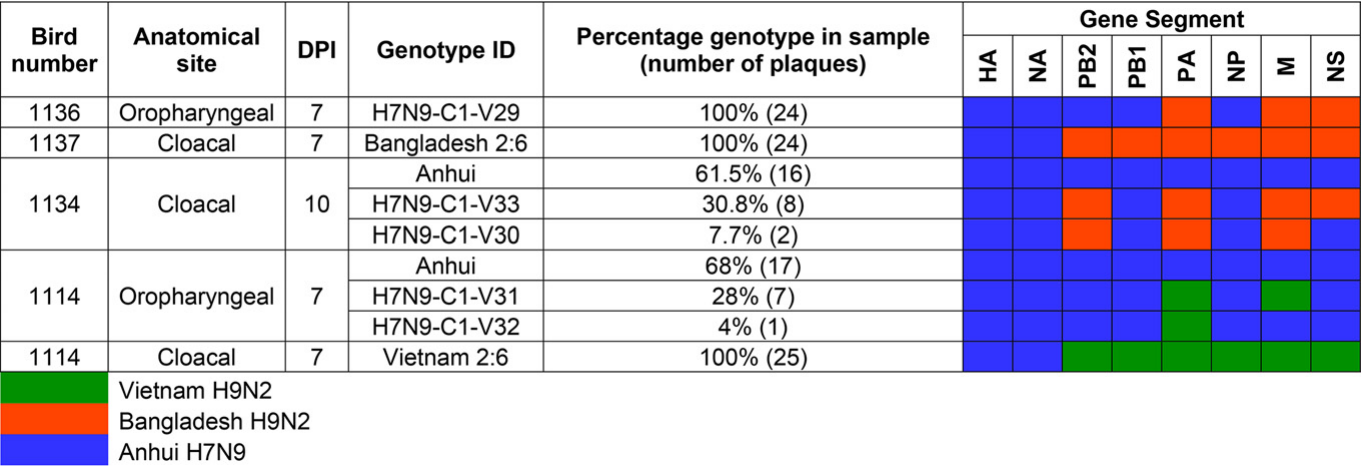

The Anhui/13 internal gene segments featured as the exclusive internal gene cassette in all plaque-recovered viruses at the C2 stage (data not shown). However, the C1 transmission stage included plaque-recovered viruses possessing genetically different internal gene segments as viable novel reassortants.

### Replication of reassortant viruses in chicken and human cells.

**FIG 4:**
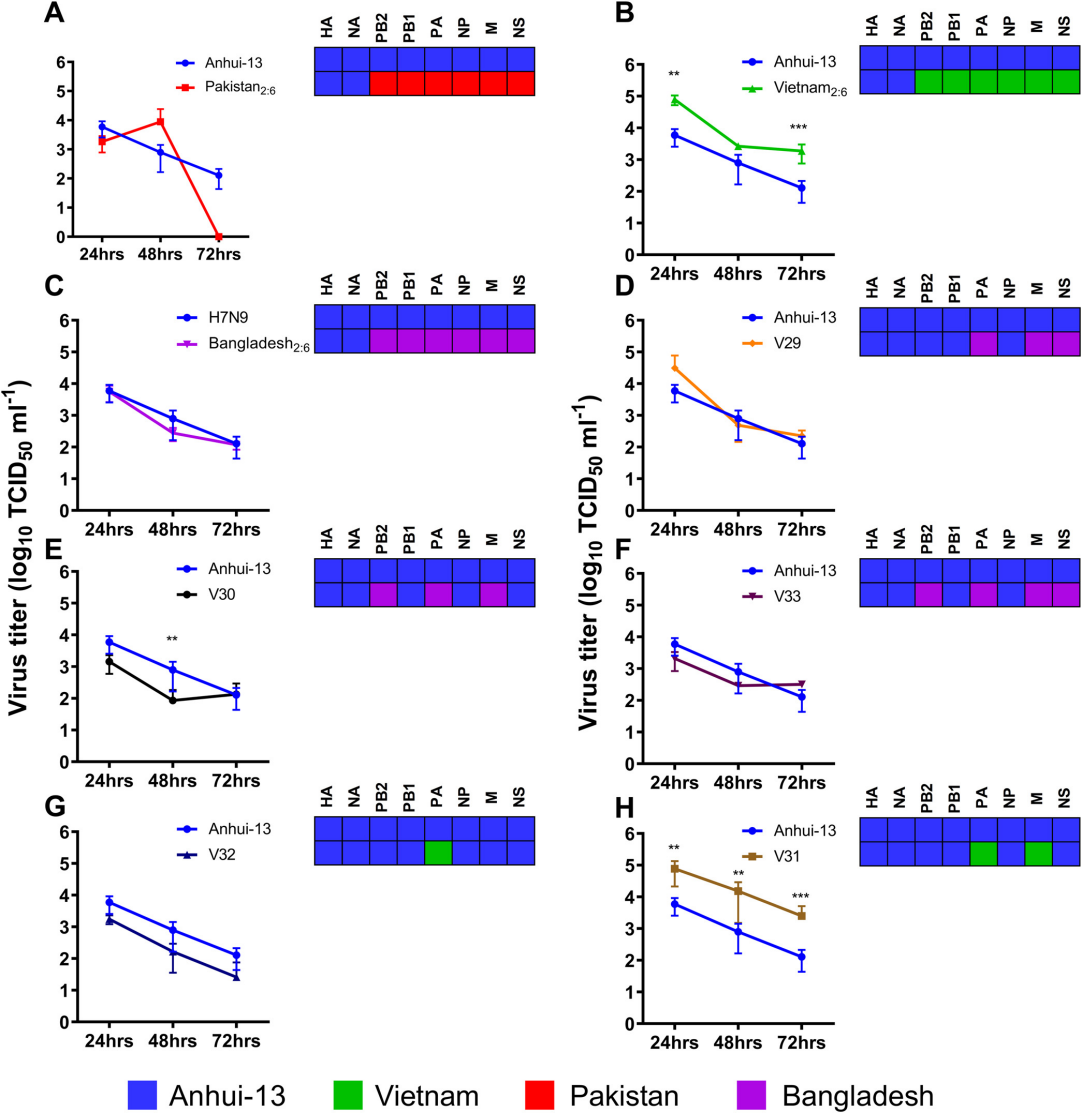
Replication of Anhui/13 H7N9, 2:6 H7N9 viruses, and the reassortant viruses in chicken DF-1 cells. (A to H) Virus replication of Anhui/13 (blue) compared to 2:6 H7N9 viruses containing the HA and NA from H7N9 and the remaining internal gene cassettes from either Pakistan (A), Vietnam (B), or Bangladesh (C) H9N2 viruses or novel reassortant viruses recovered by plaque purification from C1 chicken swabs (D to H). Cells were infected at an MOI of 0.01 with each virus, and cell supernatant was harvested at 24, 48, and 72 h postinfection. Viral titers were determined by TCID_50_. Each time point corresponds to the mean of four biological replicates with standard deviations indicated. Replication of reassortant viruses compared to parental Anhui/13 H7N9 virus is shown in panels A to H. The genotype of each reassortant virus is shown as a combination of black, red, green, and violet colors. Two-way ANOVA with multiple analyses was performed comparing every group to Anhui; **, *P* < 0.005; ***, *P* ≤ 0.001.

In addition to the three 2:6 H7N9 viruses which featured in the chicken coinfections, RG was also used to generate the five novel reassorted genotypes which emerged at the C1 stage following coinfection, namely, H7N9-C1-V29, H7N9-C1-V30, H7N9-C1-V31, H7N9-C1-V32, and H7N9-C1-V33 (Table 1). In chicken cells (DF-1) there was no statistical difference in replication titers between Bangladesh_2:6_ and Pakistan_2:6_ compared to Anhui/13 H7N9 (Fig. 4A and C). The Vietnam_2:6_ virus showed significantly higher replication titers compared to Anhui/13 at 24 h (*P* < 0.005) and 72 h (*P* < 0.001) postinfection (Fig. 4B). Among the five reassortant genotypes, only two showed statistically different replication titers compared to Anhui/13 H7N9. C1-V30 (Bangladesh PB2, PA, and M) showed reduced titers in chicken cells at 48 h postinfection (*P* < 0.005) (Fig. 4E). However, C1-V31 (Vietnam PA and M) showed significantly higher replication titers compared to Anhui/13 H7N9 (*P* < 0.005 at 24 and 48 h; *P* < 0.001 at 72 h). Further, H7N9-C1-V32 (Vietnam PA) showed comparable titers to Anhui/13 H7N9, indicating that M in combination with PA increases the replication of H7N9-C1-V31. These results suggest that internal gene segments from G1 lineage H9N2 viruses have no effect on the replication of 2:6 H7N9 viruses in chicken cells. However, some combination of G1 gene segments may reduce the replication of 2:6 H7N9 compared to Anhui/13 H7N9 virus. The internal gene segments from the Vietnam-origin (G57 genotype) H9N2 increased replication titers of 2:6 H7N9 compared to Anhui/13 H7N9 virus, which could be attributed to the M gene segment.

**FIG 5:**
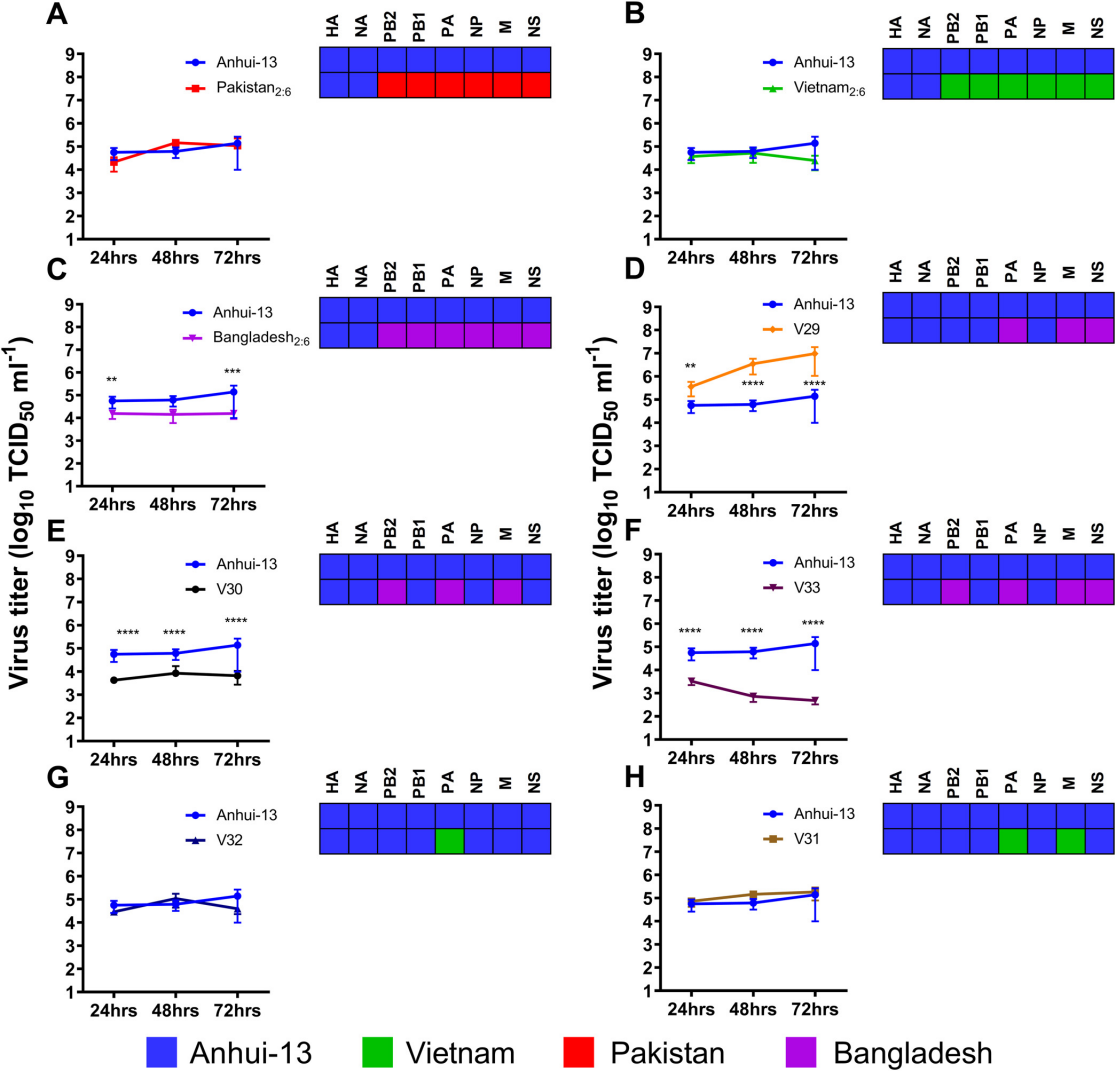
Replication of Anhui/13 H7N9, 2:6 H7N9 viruses, and the reassortant viruses in human A549 cells. (A to H) Virus replication of Anhui/13 (blue) compared to 2:6 H7N9 viruses containing the HA and NA from H7N9 and the remaining internal gene cassettes from either Pakistan (A), Vietnam (B), or Bangladesh (C) H9N2 viruses or novel reassortant viruses recovered by plaque purification from C1 chicken swabs (D to H). Cells were infected at an MOI of 0.01 with each virus, and cell supernatant was harvested at 24, 48, and 72 h postinfection. Viral titers were determined by TCID_50_. Each time point corresponds to the mean of four biological replicates with standard deviations indicated. Replication titers of reassortant viruses compared to parental Anhui/13 H7N9 virus is shown in panels A to H. The genotype of each reassortant virus is shown as a combination of black, red, green, and violet colors. Two-way ANOVA with multiple analyses was performed comparing every group to Anhui; *, *P* = 0.0113; **, *P* = 0.0012; ****, *P* < 0.0001.

In human cells (A549) Anhui/13 replicated to similar titers compared to Pakistan_2:6_, Vietnam_2:6_, and two reassortant genotypes (H7N9-C1-V31 and H7N9-C1-V32), both containing Vietnam_2:6_ internal gene segments (Fig. 5A, B, G, and H). However, one reassortant virus, H7N9-C1-V29 (Bangladesh PA, M, and NS), had significantly elevated replication titers at all time points compared to Anhui/13 (Fig. 5D). All other viruses replicated to significantly lower titers for at least one time point compared to Anhui/13 (Fig. 5D). In another human cell type (Calu-3) only H7N9-C1-V29 showed comparable replication to Anhui/13 H7N9 (Fig. S8). Together, this suggests that H7N9 virus acquiring all internal gene segments from the G1 lineage or G57 genotype of H9N2 viruses via reassortment may potentially compromise its replication competence in human cells or that replication titers may remain unaffected. Thus, reassortant 2:6 H7N9 (Pakistan_2:6_, Vietnam_2:6_, and Bangladesh_2:6_) may carry either reduced or similar zoonotic potential as Anhui/13 H7N9. However, some reassortants (possessing PA, M, and NS from Bangladesh_2:6_) exhibited increased or comparable replicative fitness in human cells (in Calu-3 or A549) compared to Anhui/13 H7N9.

### Polymerase activity of reassortant viruses in chicken and human cells.

**FIG 6:**
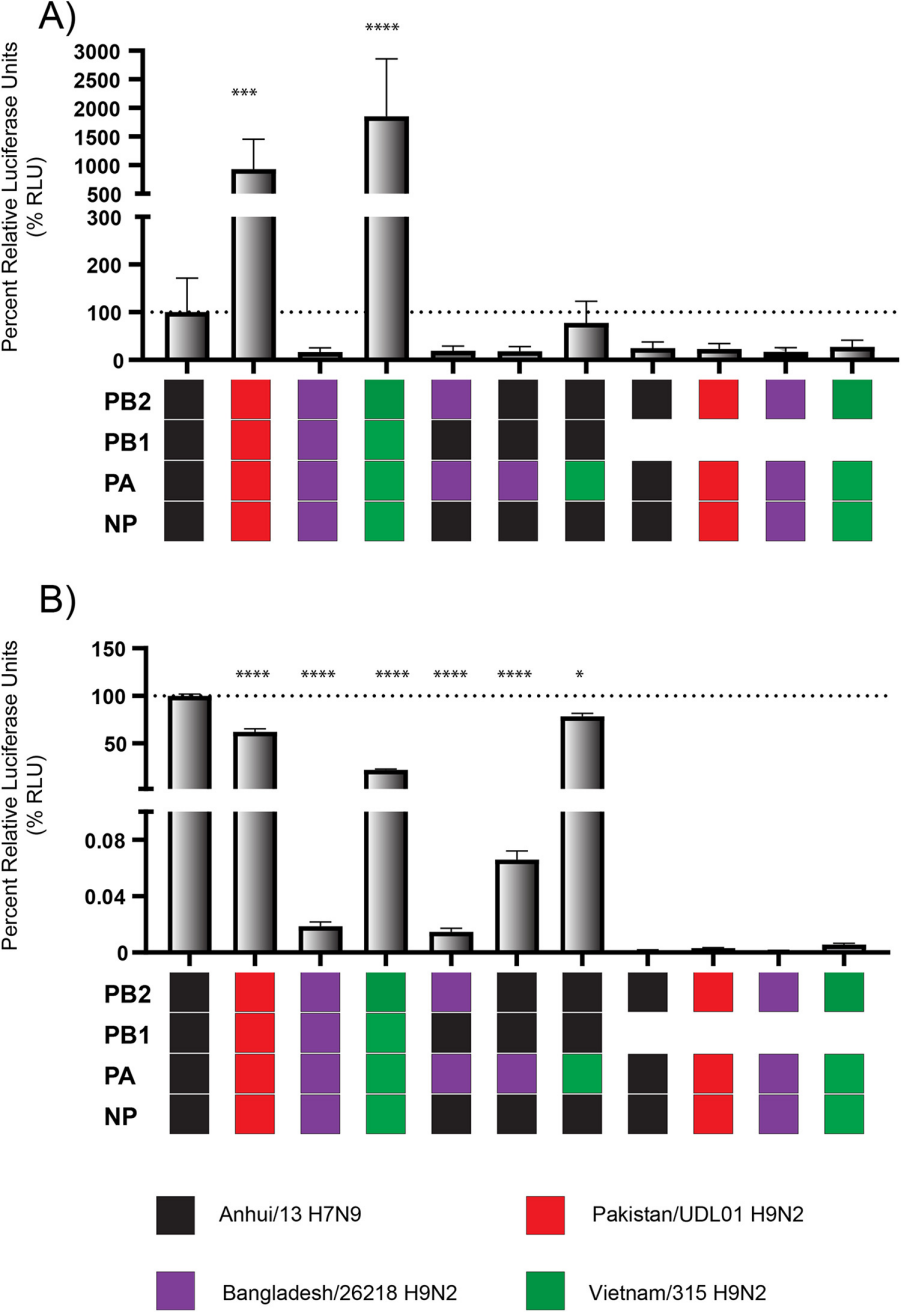
Minireplicon assays of the ribonucleoprotein (RNP) complexes of H7N9 Anhui/13 and H9N2 UDL/08, H9N2 Vietnam/315, H9N2 Bangladesh/26218, and three altered RNP combinations. (A and B) The RNP complexes were reconstituted by transfecting chicken DF-1 (A) and human HEK-293T (B) cells and incubating them at 39°C and 37°C, respectively. Luciferase activities were measured 24 h posttransfection. RNP complexes without PB1 served as negative controls. The polymerase activity of Anhui/13 H7N9 was set at 100% and the percent relative luciferase units (% RLU) was calculated. The data shown are representative of two independent experiments. Ordinary one-way ANOVA was carried out by comparison with Anhui/13 H7N9. *, *P* < 0.05; **, *P* < 0.005; ***, *P* < 0.001; ****, *P* < 0.0001.

The viral RNA-dependent RNA polymerase (vRNP) complexes of both Vietnam and Pakistan origins showed significantly higher polymerase activity than that of Anhui/13 in chicken cells (DF-1) (Fig. 6A). Comparatively, the vRNP complex of Bangladesh showed very low polymerase activity. We also compared the polymerase activity of reassortant viruses identified after plaque purification. The PA, alone or in combination with PB2 from Bangladesh and other RNPs from Anhui/13, also showed significantly lower polymerase activity in chicken cells (Fig. 6A). This observation suggested that the Pakistan and Vietnam RNP complexes are more active, while the Bangladesh RNP complex is relatively less dynamic in avian cells.

In human cells (HEK-293T), the vRNP of Anhui/13 showed statistically significantly higher polymerase activity than the vRNPs of Pakistan, Bangladesh, and Vietnam (Fig. 6B). Among the H9N2 vRNP complexes, Pakistan exhibited the highest polymerase activity, followed by Vietnam, with the vRNP of Bangladesh exhibiting the lowest polymerase activity. The PA alone, or in combination with PB2 on Anhui/13 H7N9 background, also showed reduced polymerase activity in human cells. This observation suggested that the vRNP of Anhui/13 is more active in human cells, followed by Pakistan and Vietnam, while Bangladesh is relatively less dynamic in human cells.

## DISCUSSION

The A/Anhui/1/2013-lineage H7N9 LPAIV emerged in China in 2013 and continued to reassort with local enzootic H9N2 viruses (2, 28). We modeled potential outcomes of reassortment events between H7N9 and the H9N2 viruses enzootic in poultry in the wider Asian geographical regions. The G1 lineage is prevalent in birds in the Indian subcontinent, the Middle East, and Africa, whereas BJ94-like viruses are enzootic in China and neighboring countries. To test the propensity of H7N9 acquiring internal genes from other cocirculating H9N2 viruses, three 2:6 H7N9 RG viruses were generated which contained external HA and NA genes of the A/Anhui/1/2013-lineage H7N9 and internal gene segments from three different enzootic H9N2 AIVs. Chickens infected with these three reassortants showed clear differences in their replication and transmissibility. Both Bangladesh_2:6_ and Pakistan_2:6_ contained G1 lineage internal gene cassettes but differed in their transmission efficiency, with the former clearly failing to transmit (i.e., 0% transmission to contacts), while the latter demonstrated 67% transmission efficiency to contacts. While original H9N2 Pakistan virus transmitted efficiently (100%) and rapidly to contact chickens as observed in previous studies (36–38), coupling of its internal genes with the HA and NA from Anhui/13 reduced the transmission efficiency, despite a higher polymerase activity in chicken cells for the Pakistan vRNP complex compared to that of Anhui/13 (34). It is likely that internal gene cassettes from H9N2 viruses akin to Bangaldesh_2:6_ and Pakistan_2:6_ would be unable to establish sustained circulation in chickens and would therefore not contribute to any prolonged changes in H7N9 epidemiology. The reduced infectivity and transmissibility of the 2:6 H7N9 viruses could be due to incompatibilities between the surface glycoproteins and the internal genes, through either protein or RNA interactions affecting packaging signals. Indeed, packaging signal incompatibilities have been observed to hinder the fitness of reassortment viruses generated between China-origin H7N9 and human H3N2 viruses (39) and clearly plays an important part in the emergence of novel genotypes through reassortment (40). In contrast to the two 2:6 H7N9 reassortants with G1 H9N2 internal genes (Pakistan_2:6_, Bangaldesh_2:6_), Vietnam_2:6_ most closely resembled the ability of Anhui/13 to both shed (notably from the cloacal tract) and transmit successfully at 100% efficiency. The close phylogenetic similarity of internal genes originating from Anhui/13 (G57 origin) and the Vietnam H9N2 compared to those of the other two G1 lineage H9N2 AIVs may at least partly explain this observation (Fig. S2).

To investigate the contribution of internal gene segments on viral fitness, we performed three *in vivo* coinfection experiments in chickens. We used a 1:1 mix of the two parental viruses in a synchronized inoculation; while this is an unlikely scenario in nature, it provides a standardized system to allow the direct comparison of internal gene fitness and gene segment contribution. We have previously generated a large range of reassortant viruses *in vivo* between Anhui/13 and the Pakistan H9N2 AIV, where the progeny included H9N2 and a novel H9N9 virus subtype, despite 1,000-fold lower titers of Pakistan virus in the initial mixed inoculum (34). The current study featured coinfections of the 2:6 H7N9 reassortants with the unaltered prototype H7N9 (Anhui/13), where any reassorted viable progeny viruses would be constrained to possess the Anhui/13 HA and NA external genes (i.e., retaining the H7N9 subtype), but with the potential to include novel genotypes involving the internal gene cassettes of G57-origin (Anhui/13), G1-like, or BJ94-like lineage H9N2 AIVs. Importantly, the *in vivo* dynamics of the range of potential new reassortants was assessed through to the C2 transmission stage, at which point the shedding of exclusively unaltered Anhui/13 was detected following the three initial coinfections. We therefore demonstrated the favorable combination of the Anhui/13 external gene segments along with all six of its G57-origin internal gene segments, which at the C2 stage had successfully outcompeted any new genotypes *in vivo*. As the contact chickens were cohoused, transmission between contacts, rather than from direct infection to contacts, is possible. This represents the possibility of enrichment of a particular genotype through stochastic events rather than by a true fitness advantage. Despite these factors, these data suggest that the identification of novel influenza A virus threats to animal and human health may be at least partly dependent on understanding the replicative advantages conferred by internal gene cassettes such as those of G57-origin.

Despite an ultimate selective advantage for Anhui/13 internal gene segments, a range of H9N2-origin internal gene segments were identified as transient H7N9 viable genotypes during shedding at the Di and C1 stages. The five novel H7N9 genotypes all possessed at least one of the three polymerase gene segments of either Vietnam or Bangladesh origin, but there was no detectable transmission of any of the Pakistan-origin internal gene segments (Fig. 3 and Table 1). The previously observed viral progeny from the Anhui/13 and H9N2 Pakistan coinfections frequently included the H9 HA gene (34), which again suggested that gene incompatibilities existed between the H7 HA gene and Pakistan-origin internal gene segments in the current study. The relative abundance and range of transient novel genotypes was relatively low for all coinfections, despite the administration of equal infectious levels of both viruses in the three inocula. However, the level of reassortment was greatest for the BJ94-like virus (Vietnam_2:6_) coinfection, where a higher abundance of Vietnam-origin gene segments was also detected in the cloacal swab samples. This observation was consistent with the increased cloacal shedding observed in the single-virus infections with Vietnam_2:6_. We identified the PB2, NP, and M gene segments from the BJ94-like lineage virus to be abundantly shed from the oropharyngeal cavity of the Di chickens at the later time point. Interestingly, the PB2, NP, and M gene segments have also been identified as the key virulence genes for Anhui/13 in mice (24).

In the case of the coinfections which featured the H9N2 G1-like lineage internal gene segments (Pakistan_2:6_ and Bangladesh_2:6_), the G1-origin NP was absent in all Di oropharyngeal samples, even as early as 1 day postinfection, indicating that the Anhui/13 NP is immediately selected in preference to G1-like NP gene segments. Interestingly, in the field, sequence analysis indicated that H7N9 has inherited NP genes from cocirculating H7N9 viruses instead of H9N2 viruses, despite frequent cocirculation of H9N2 viruses in poultry in China (41). Moreover, mutational analysis indicated divergent evolutionary pathways between NP genes from H7N9 and H9N2 viruses (41). Furthermore, the 2014 human H5N6 virus isolate first reported in the Sichuan Province of China contained an NP gene segment with 99% similarity to that in contemporary H7N9 and H10N8 viruses (41). Together, these observations support our findings that the NP gene segment from Anhui/13 is more optimal in chickens than the NP of the G1-lineage viruses and may readily replace other NP segments during reassortment events in the field. However, no significant increase in polymerase activity was associated with Anhui/13-origin NP in chicken cells in the current study (Fig. S7) or, indeed, previously (34). These observations indicate that the role of Anhui/13-NP in reassortment merits further investigation.

Multistep replication analysis in chicken cells showed that the reassortants Vietnam_2:6_ and H7N9-C1-V31 replicated at significantly higher titers than Anhui/13. This increased replication is partially attributed to the higher polymerase activity of Vietnam-origin internal genes and implied that H7N9 reassortment with G57-lineage H9N2 viruses may occur in chickens to produce viable viruses possessing an increased replicative fitness in this host. The multistep replication analysis in human cells showed significantly increased replication associated with Anhui/13 H7N9 compared to most of the reassortant viruses, which also correlated with the higher polymerase activity of Anhui/13 in human cells. Moreover, *in silico* analysis of the cumulative number of humanizing polymorphisms present in every potential reassortment virus used in this study revealed that the wild-type (WT) Anhui/13 still had the greatest number, and thus all other potential reassortants posed a lower risk to human health. However, the reassortant viruses H7N9-C1-V29 and H7N9-C1-V31 (containing Bangladesh- and Vietnam-origin H9N2 internal genes, respectively) showed increased and comparable replication to Anhui/13 in human cells, respectively. Thus, in the case of both G57-like and G1-like lineage H9N2 viruses reassorting with H7N9 viruses, there is a possible outcome which includes the emergence of novel viruses with a potentially increased threat to human health. However, the human threat of these viruses was only assessed *in vitro*, and investigation using animal models *in vivo* would be required to appreciate the true public health risk.

Among the five reassortant viruses recovered from three C1 chickens, three viruses originated from the Bangladesh_2:6_, while two viruses originated from Vietnam_2:6_ coinfected groups. Interestingly, two of these C1 chickens harbored multiple genotypes in the swab samples. In addition, all three chickens also shed a genetic constellation which differed between the oropharyngeal and cloacal cavity from the same bird. This observation was surprising because transmission is often considered to represent a bottle neck to viral (including influenza A virus) diversity, with only limited virus genotypes being transmitted to successfully establish infection in the new host due to the triggering of local immune responses (42–44). However, the effect of the bottle neck may vary, depending on species and transmission route (45). In avian hosts, the replication and dissemination of progeny AIV occurs via both the respiratory and enteric tracts, suggesting that two anatomically separate bottle necks may function simultaneously in infected birds as has been hypothesized previously (46, 47).

Several studies have investigated the consequences of H7N9 and H9N2 virus reassortment by generating a panel of viruses by reverse genetics and focused on potential zoonotic outcomes using mouse models of infection (23, 48). We differed by investigating the consequences of mixed infections of H7N9 subtypes in chickens, arguably the species in which novel reassortant viruses are most likely to be generated and maintained, with subsequent potential spillover to humans. In addition to our previous study (34), another investigation has described *in vivo* reassortment in chickens between H7N9 and H9N2 viruses (49). The authors adopted a similar approach to our previous study (34) by using whole WT AIVs, with the corresponding WT HA and NA gene segments (i.e., H7, H9, N9, and N2) (49). They reported a greater range of reassortment between two A/Anhui/1/2013-lineage H7N9 viruses and G57-like, G44-like, and G1-like H9N2 viruses in comparison to our current study. However, both studies concurred in that emerging reassortants became rapidly outcompeted by the H7N9 WT variants during chicken transmission (49). The studies differed concerning the detail of reduced transmissibility of reassortant viruses, whereby we previously showed that H7N9 WT coinfection with a more contemporary G1-like virus generated H9N2 and H9N9 reassortants which transmitted efficiently in chickens (34). In this study, we focused on the role of the internal genes, removing the confounder of the HA and NA gene segments, and compared the H9N2 internal gene cassettes in isolation. Importantly, our coinfection approach provided an opportunity for replicative competition between internal genetic segments. In contrast to Su et al. (49), we observed that H7N9 viruses possessing G1-like and BJ94-like features are capable of efficient transmission in chickens but were rapidly outcompeted when competition was provided by H7N9 coinfection. To our knowledge, we have reported the first description of coinfection and transmission outcomes resulting from different internal gene constellations from H7N9 and several different H9N2 viruses.

While widespread vaccination of poultry in China has largely controlled human cases of H7N9 (41), this was implemented following the emergence of an H7N9 HPAIV derivative (50). H7N9 has been sporadically detected in poultry and the environment in farms and live bird markets across different provinces of China (Fujian, Guangdong, and Henan) between January 2020 and October 2021 (16), suggesting continued circulation in Chinese poultry. Our data suggested that while the Anhui/13 internal gene cassette is optimal for transmission of the A/Anhui/1/2013-lineage H7N9, reassortment with other H9N2 viruses can occur in chickens to produce viable viruses capable of transmission. In addition, these viruses may have altered biological properties which may elevate their zoonotic threat.

## MATERIALS AND METHODS

### Ethics statement.

All animal studies and procedures were carried out in strict accordance with European and United Kingdom Home Office legislation. As part of this process, the *in ovo* and *in vivo* work was subject to scrutiny and approval by the Animal Welfare Ethical Review Board (AWERB) at The Pirbright Institute (TPI), and the Animal and Plant Health Agency (APHA), Weybridge, UK, respectively. Any infected poultry which began to display severe clinical signs were euthanized and were recorded as a mortality. UK regulations categorize the H7N9 LPAIV as a Specified Animal Pathogens Order (SAPO) 4 and Advisory Committee on Dangerous Pathogens (ACDP) hazard group 3 pathogen because it is a notifiable animal disease agent and presents a zoonotic risk. All laboratory and infectious work with H7N9 specimens, including infected poultry and eggs, was done in licensed ACDP3/SAPO4 laboratories of TPI or APHA.

### Influenza A viruses prepared by reverse genetics and their propagation.

The nucleotide sequences of the different gene segments of H7N9 and all H9N2 viruses were retrieved from publicly accessible databases, namely, the Global Initiative on Sharing All Influenza Data (GISAID; https://www.gisaid.org/) or the National Center for Biotechnology Information (NCBI; https://www.ncbi.nlm.nih.gov/). Gene segments were synthesized using GeneArt (Thermo Fisher Scientific) and subcloned into the pHW2000 vector by restriction enzyme-dependent (51), or restriction enzyme- and ligation-independent (52, 53), cloning techniques. The four influenza viruses used in the study were prototype A/Anhui/1/2013-lineage H7N9 A/Anhui/1/13 (abbreviated to Anhui/13, GISAID accession no. EPI_ISL_138739) and three H9N2 viruses which supplied the six internal gene segments for 2:6 reassortment with the hemagglutinin (HA) and neuraminidase (NA) of Anhui/13, namely, A/chicken/Pakistan/UDL-01/2008 (NCBI accession no. CY038455), A/Environment/Bangladesh/26218/2015 (NCBI accession no. KY635657), and A/chicken/Vietnam/H7F-14-BN4-315/2015 (GISAID accession no. EPI_ISL_327772). The 2:6 reassorted viruses were rescued by reverse genetics (36, 54) and propagated in 10-day-old specific-pathogen-free (SPF) embryonated chicken eggs at 37°C for 72 h. The Madin-Darby canine kidney (MDCK) and human embryonic kidney (HEK) 293T cells (ATCC) were maintained with Dulbecco’s modified Eagle’s medium (DMEM) (Sigma) supplemented with 10% fetal calf serum (FCS) (Sigma), 100 U/mL penicillin, and 100 μg/mL streptomycin (Gibco) at 37°C under 5% CO_2_ (vol/vol).

### 50% Egg infectious dose (EID_50_) determination.

The egg infectious dose of the viruses was calculated by making 10-fold serial dilutions of the viruses. Then, 100 μL of each dilution was inoculated in a group of 6 eggs, and the eggs were incubated at 37°C for 72 h (55). Allantoic fluid was harvested from all the inoculated eggs and tested for the presence or absence of virus by the hemagglutination assay (55). The EID_50_ titer was calculated by the method described by Reed and Muench (56). The viruses were aliquoted and stored at −80°C till further use.

### AIV infections of chickens.

The specific-pathogen-free (SPF) chickens of Rhode Island red variety (procured from the National Avian Research Facility (NARF), Roslin Institute, Edinburgh, UK) at 3 weeks of age were used in the experiment. For the single-virus infections, three groups of six chickens were infected intranasally (i.n.) with 200 μL of inoculum (applied across both nares of each directly infected (Di) chicken), containing 1 × 10^8^ EID_50_ of the three 2:6 reassortant viruses (i.e., with Pakistan-, Bangladesh-, or Vietnam-origin internal genes), and a fourth group of six Di chickens was similarly infected with 1 × 10^8^ EID_50_ of Anhui/13. Contact chickens (*n* = 18), referred to as C1 (C denotes contact), were introduced for cohousing with Di chickens at 1-day postinfection (dpi). For the coinfections, Di chickens (*n* = 6/group) were coinfected i.n. with 200 μL of inoculum containing a mixture of the 1 × 10^8^ EID_50_ of Anhui/13 and 1 × 10^8^ EID_50_ of the reassortant 2:6 H7N9 viruses. C1 chickens (*n* = 6/group) were introduced for cohousing at 1 dpi. When transmission to 50% of the C1 chickens had occurred, determined by testing in real time, all Di chickens were removed from cohousing by culling, and the second group of contact chickens, referred to as C2 (C2 denotes 2nd contact), were added for cohousing at 4 or 5 dpi. For single-virus and coinfected chickens, oropharyngeal and cloacal swabs were collected daily in virus transport medium (VTM) (34) from all the directly infected chickens and contact chickens until 14 dpi. Chickens were monitored for clinical signs up to three times daily. Any surviving chickens were humanely euthanized by overdose of pentobarbitone at 14 dpi, with blood collected by terminal heart bleeds. Time points relating to the C1 and C2 contact chickens may at times be referred to as days postcontact (dpc), as appropriate.

### Serology.

Sera derived from clotted blood were heat-inactivated at 56°C for 30 min. Seroconversion in the infected chickens was identified via the hemagglutination inhibition (HI) test (55), using the homologous inactivated Anhui/13 virus as antigen titrated to 4 hemagglutination units.

### Avian influenza virus screening RT-qPCR.

RNA was extracted from VTM swab fluids by robotic extraction using the RNeasy RNA extraction kit (Qiagen), as described previously (57, 58). For initial screening purposes, all extracted RNA specimens were tested by the M-gene RT-qPCR using the primers and probe, as described previously (59, 60). A 10-fold dilution series of titrated H7N9 RNA was used to calculate viral titers, which are displayed as relative equivalency units (REUs) (34).

### H7N9/H9N2 gene-specific RT-qPCR.

Segment-specific primer and probes for Pakistan, Bangladesh, and Vietnam viruses were designed to distinguish between the H7N9- and H9N2-origin internal gene segments, as described previously (34, 57). Briefly, each segment-specific assay consisted of a pair of conserved primers which bind to identical sequences which are shared within each segment of Anhui/13 and an H9N2 virus. These conserved primers generated amplicons which were distinguished as regards their viral origin by using probes which were targeted to strain-specific nucleotide sequences within the amplicons. Each probe was 5′-labeled with a different fluorophore, namely, FAM and HEX for Anhui/13 and H9N2-origin segments, respectively (Table S1). All assays were adapted to the one-step RT-PCR kit (Qiagen) reaction chemistry, as described previously, and used the same thermocycling conditions (34). Each segment-specific RT-PCR was compared for its analytical sensitivity to demonstrate equivalent performance of the assays (Fig. S6). RNA from oropharyngeal and cloacal samples was analyzed by both RT-qPCR assays to calculate the relative percent abundance (origins) of each gene segment.

### Plaque purification.

MDCKs in 6-well-plates were infected with a 10-fold serial dilution of a swab suspension made in 500 μL of DMEM and incubated for 1 h at 37°C. The infection media were removed, and 2 mL of overlay medium containing 1 μg/mL of TPCK (tosylsulfonyl phenylalanyl chloromethyl ketone) trypsin (Sigma) with 2% agar was added, inverted, and incubated at 37°C with 5% CO_2_ for 4 days or until plaques were formed. Plaque purification was performed in quadruplicate in six-well plates. Discrete plaques were identified and harvested into 200 μL of serum-free medium using a pipette tip pushed through the layer of agar until contact with the surface of the plate was made. RNA was extracted from the medium plaque suspensions by robotic methods described previously (57, 58).

### Replication analysis of reassortant viruses.

Replication analysis of the reassortant viruses was assessed in DF-1 cells (chicken embryo fibroblast), A549 (human alveolar basal epithelial), and Calu-3 (human lung) cells. Replication analysis was performed using 12-well plates in quadruplicate. Cells at 90% confluence were infected with 0.001 multiplicity of infection (MOI), calculated based on cell numbers at confluence for each cell line, of respective viruses in DMEM containing antibiotics and 0.3% bovine serum albumin (BSA) containing 0.5 μg/mL of TPCK trypsin (Sigma). The cell supernatant was harvested at 24, 48, and 72 h postinfection and titrated by 50% tissue culture infective dose (TCID_50_) in MDCK cells (56).

### Minireplicon assay.

The polymerase activity of different vRNP combinations was assessed *in vitro* by plasmid-based reporter gene expression in 24-well plates (61). Chicken DF-1 cells and human HEK-293T were transfected with expression plasmids for different RNP combinations prepared from the Anhui/13, Pakistan, Bangladesh, and Vietnam progenitor viruses by using Lipofectamine 2000 (Invitrogen), as per the manufacturer’s instructions. Then, 80 ng of pCAGGS plasmid encoding PB2 and PB1, 40 ng of pCAGGS plasmid encoding PA, and 160 ng of pCAGGS plasmid encoding NP were cotransfected with 40 ng of a renilla luciferase pCAGGS expression plasmid and 80 ng of a pCk-PolI-Firefly plasmid. The cells were incubated for 24 h at 37°C (for HEK-293T) or 39°C (DF-1), and luciferase activity was measured by using the Dual-Glo luciferase assay system (Promega). The polymerase activity was calculated by normalizing firefly luciferase activity relative to the renilla luciferase activity.
