# Supplementary material for: The Origin of Internal Genes Contributes to the Replication and Transmission Fitness of H7N9 Avian Influenza Virus
Source: J Virol. 2022 Nov 7;96(22):e01290-22. doi: 10.1128/jvi.01290-22 (PMC9683025; doi:10.1128/jvi.01290-22)
Supplement: Supplemental file 1 — Fig. S1 to S8 and Tables S1 and S2. Download jvi.01290-22-s0001.pdf, PDF file, 1.0 MB [file jvi.01290-22-s0001.pdf]

Supplementary materials

The origin of internal genes contributes to the replication and transmission fitness of H7N9 avian influenza virus

Joe James[1¶], Sushant Bhat[2¶], Sarah K. Walsh[1], H. M. Thusitha. K. Karunarathna[2], Jean-Remy Sadeyen[2], Pengxiang Chang[2], Joshua E. Sealy[2], Sahar Mahmood[1], Benjamin C. Mollett[1], Marek J. Slomka[1], Sharon M. Brookes[1], and Munir Iqbal[2*]

[1]Animal and Plant Health Agency, Weybridge KT15 3NB, UK

[2]Avian Influenza Group, The Pirbright Institute, Woking GU24 0NF, UK

[¶] Contributed equally

* Corresponding author: Professor Munir Iqbal: munir.iqbal@pirbright.ac.uk

Supplementary materials

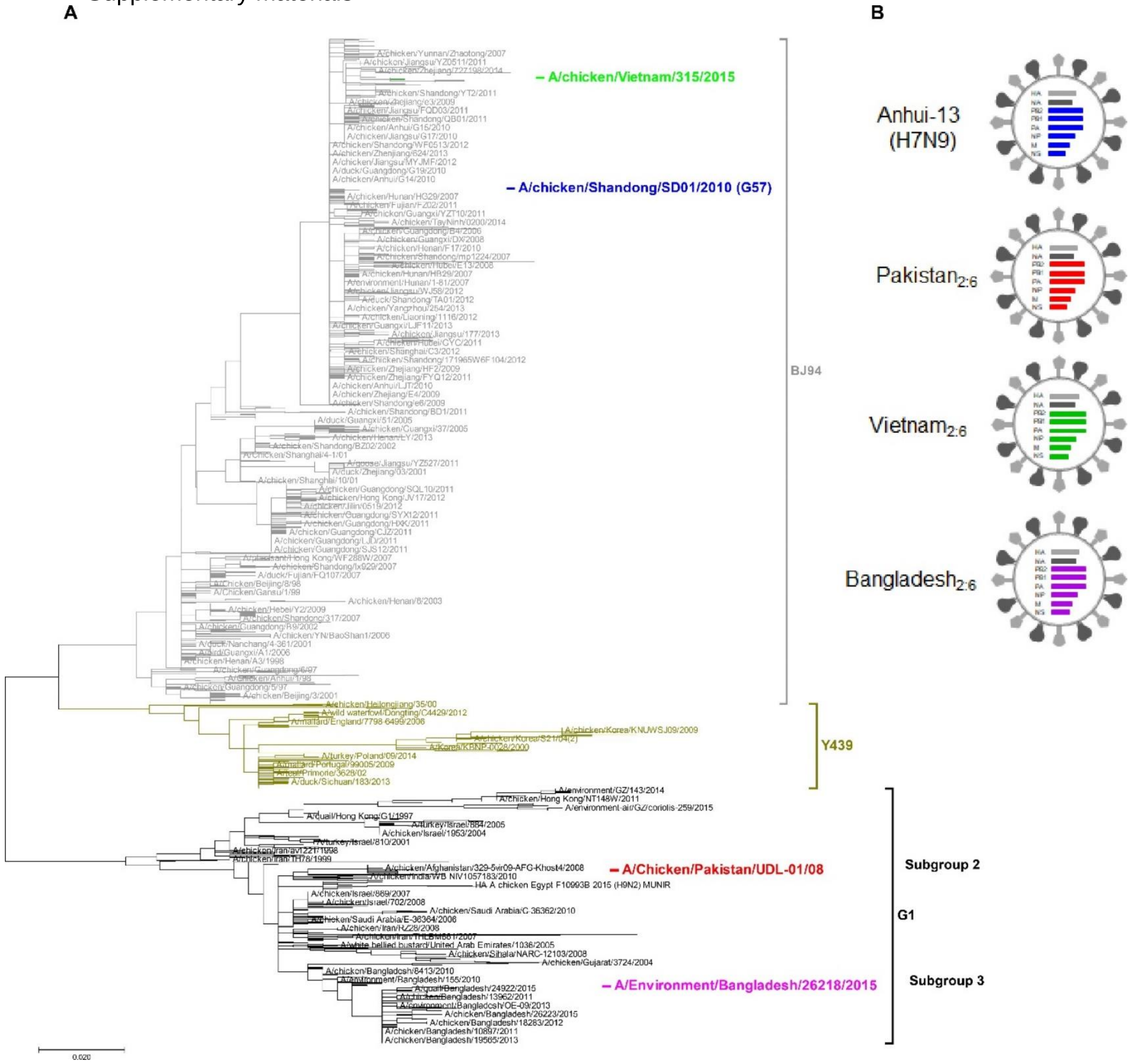

Supplementary Figure 1. Phylogenetic analysis of Haemagglutinin (HA) genes of H9N2 AIVs and a schematic of the viruses used in this study. (A) Phylogenetic relationship of the HA genes of the H9N2 AIVs which were used to generate the 2:6 reassortant H7N9 viruses by reverse genetics (RG). (B) The identity of the viruses is shown in different colours; red, Pakistan; green, Vietnam; purple, Bangladesh; blue, Anhui/13 or its ancestral H9N2 virus (G57). Phylogenetic tree was generated by maximal-likelihood analysis using H9Nx sequences downloaded from GISAID Epiflu database.

Supplementary materials

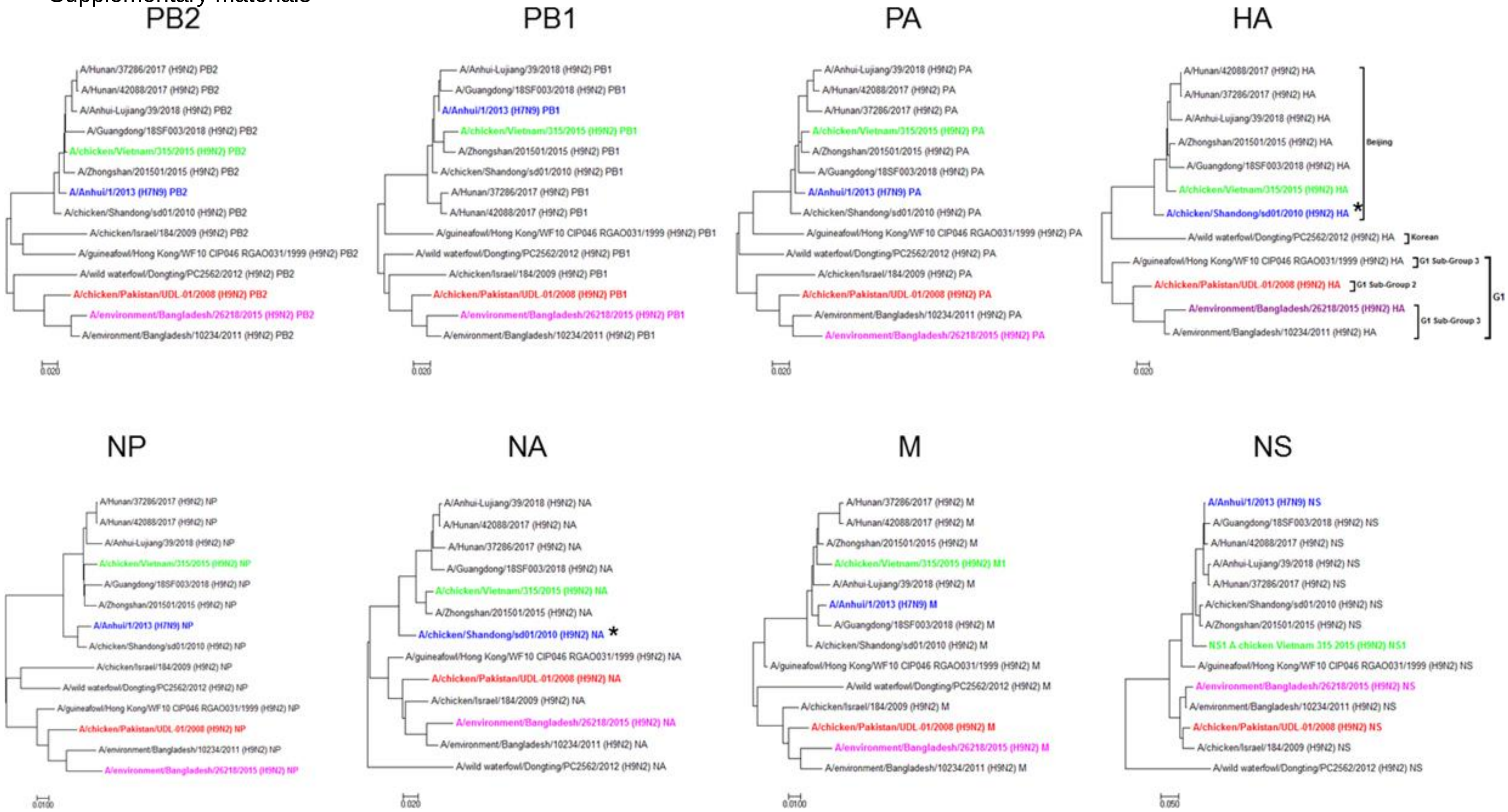

Supplementary Figure 2. Phylogenetic analysis of the internal genes of the H9N2 AIVs which provided internal genes for this study. A maximal likelihood phylogenetic tree of all genes of the viruses including several reference viruses representative of the different dominant clades. The phylogenetic location of the viruses used in the study is shown in different colours; blue, Anhui; red, Pakistan; green, Vietnam; purple, Bangladesh. * HA and NA gene segments shown were from the closest related H9N2 virus which naturally donated its internal gene cassette to produce the prototype H7N9 Anhui/13.

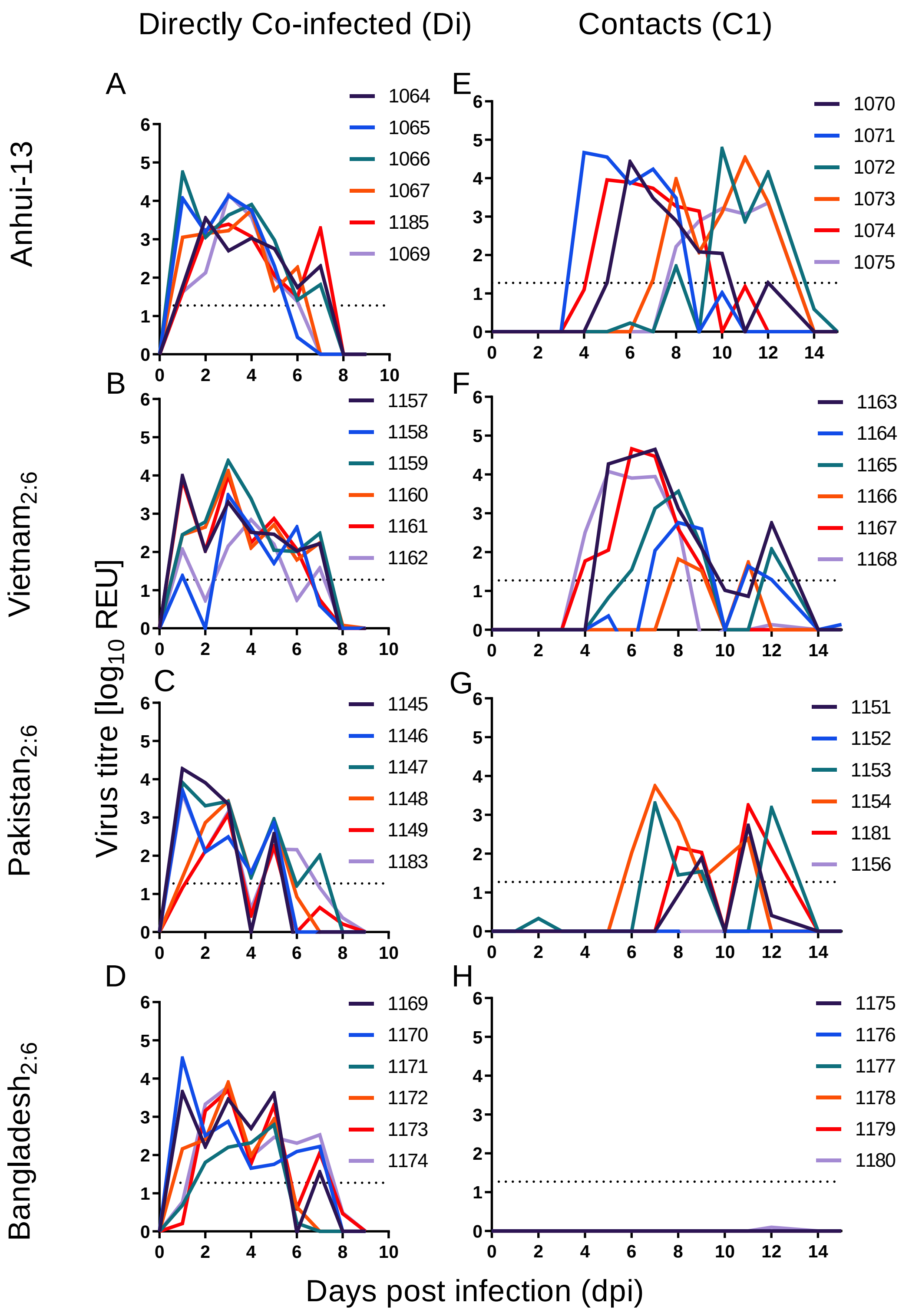

Supplementary Figure 3. Viral shedding and transmission of Anhui/13 or reassorted 2:6 H7N9 viruses possessing different H9N2 internal gene segments in individual chickens. Influenza virus titres from oropharyngeal swabs of chickens (n=6 per group) infected with $1x10^8$ EID_{50} of Anhui/13 (A) or 2:6 H7N9 viruses possessing the internal gene segments from either Vietnam (B), Pakistan (C) or Bangladesh (D) H9N2 viruses. Oropharyngeal shedding titres from chickens placed in-contact with infected groups at 1 dpi (E-H). Viral shedding displayed as relative equivalency units (REUs) based on M-gene RT-qPCR derived from a standard curve (dilution series) of viral RNA from titrated Anhui/13. Each coloured line represents shedding from an individual chicken. Dotted horizontal line indicates the positive cut-off at a Ct value of 36.

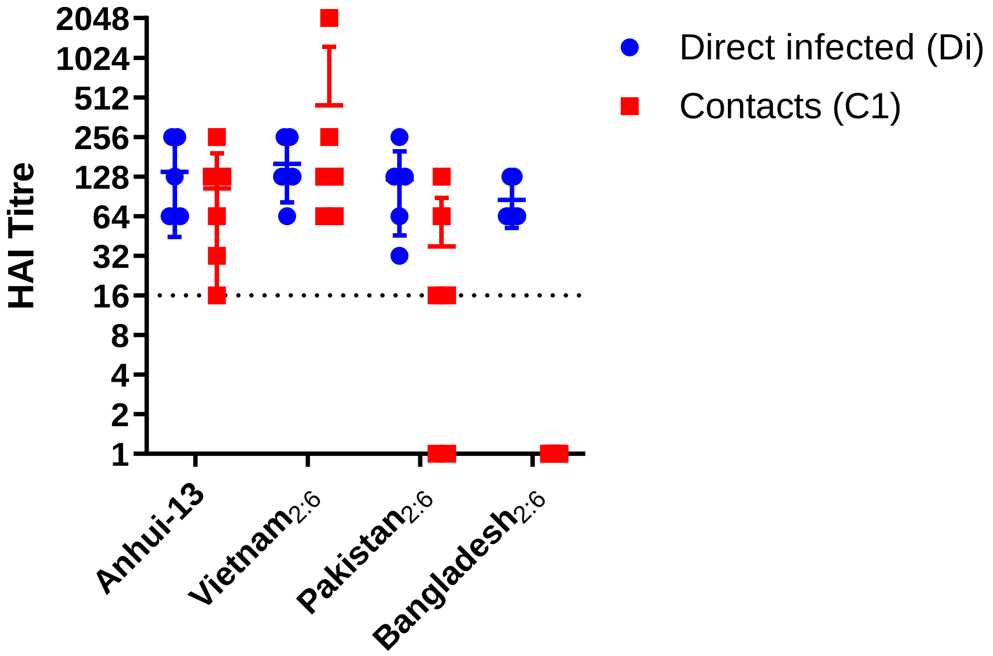

Supplementary Figure 4. H7N9 seroconversion of chickens infected with Anhui/13 or 2:6 H7N9 viruses possessing different H9N2 internal gene segments. Sera from Di and C1 chickens infected with Anhui/13 or each of the different 2:6 H7N9 viruses (n=6 per group), collected at 14 dpi and tested by the HI assay using the H7N9 Anhui/13 antigen. Individual titres with geometric mean +/- SD are shown. Dotted line represents the HI positive cut-off threshold titre at 1/16.

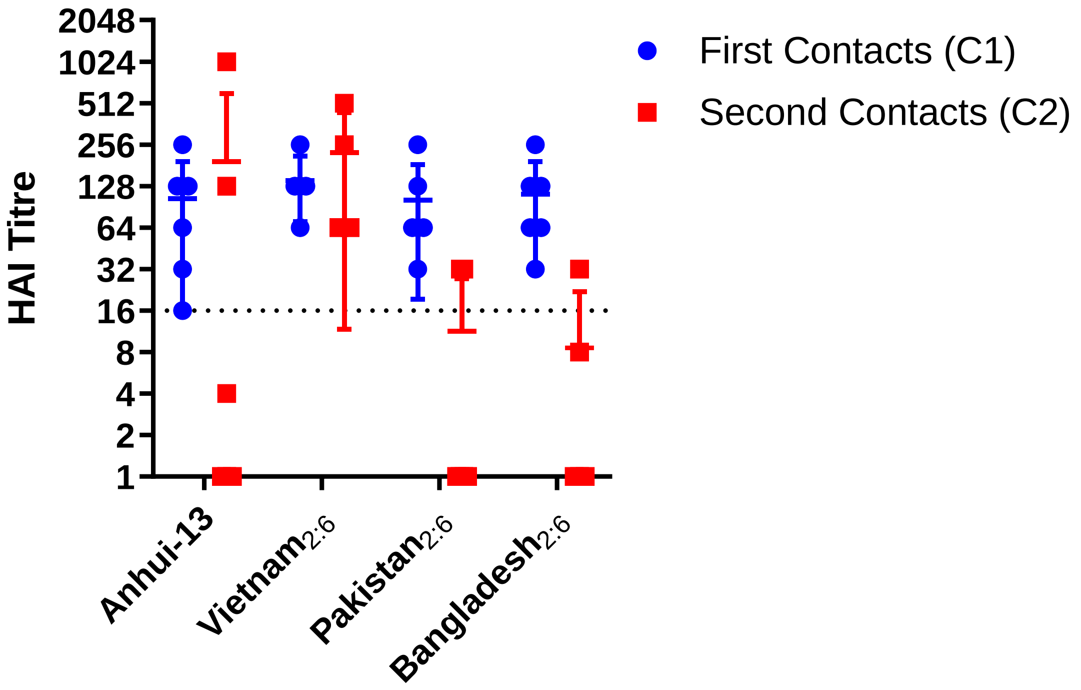

Supplementary Figure 5. Seroconversion of chickens placed in-contact with chickens co-infected with Anhui/13 and an individual 2:6 H7N9 virus possessing different internal gene segments. Seroconversion from C1 and C2 chickens was measured in a transmission chain from chickens co-infected with Anhui/13 or each of the different 2:6 H7N9 viruses, with six chickens per group. Sera were collected at 14 dpi and tested HI using H7N9 Anhui/13 antigen. Individual titres with geometric mean +/- SD are shown. Dotted line represents the HI positive cut-off threshold titre at 1/16.

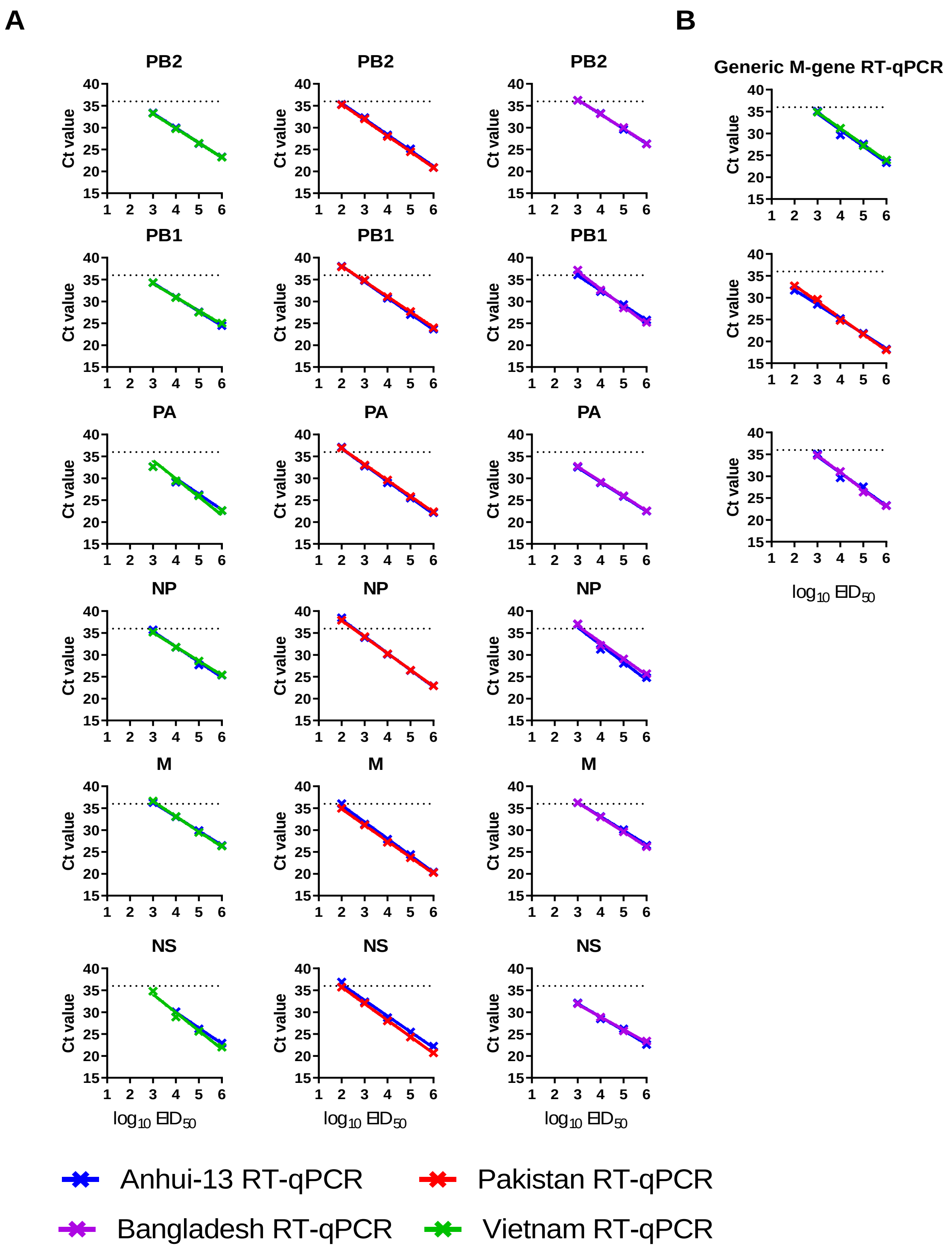

Supplementary Figure 6. Comparative sensitivity of the gene segment specific RT-qPCRs. Influenza segment and virus specific RT-qPCRs developed for Anhui/13 (HEX fluorescence) and the 2:6 reassorted H7N9 viruses with internal genes from Vietnam, Pakistan or Bangladesh H9N2 AIVs (FAM florescence). The pairs of assays were tested for equivalence against 10-fold serial dilutions of viral RNA extracted from each virus, starting at $1x10^6$ EID_{50}, and are shown for (A) the segment-specific RT-qPCRs which distinguish the viral origin, and for the (B) generic M-gene RT-qPCR which served to quantify the total viral shedding during the *in vivo* experiments. Ct values are plotted against virus titre, lines of best fit are displayed. Dotted lines represent the positive cut-off at Ct 36.

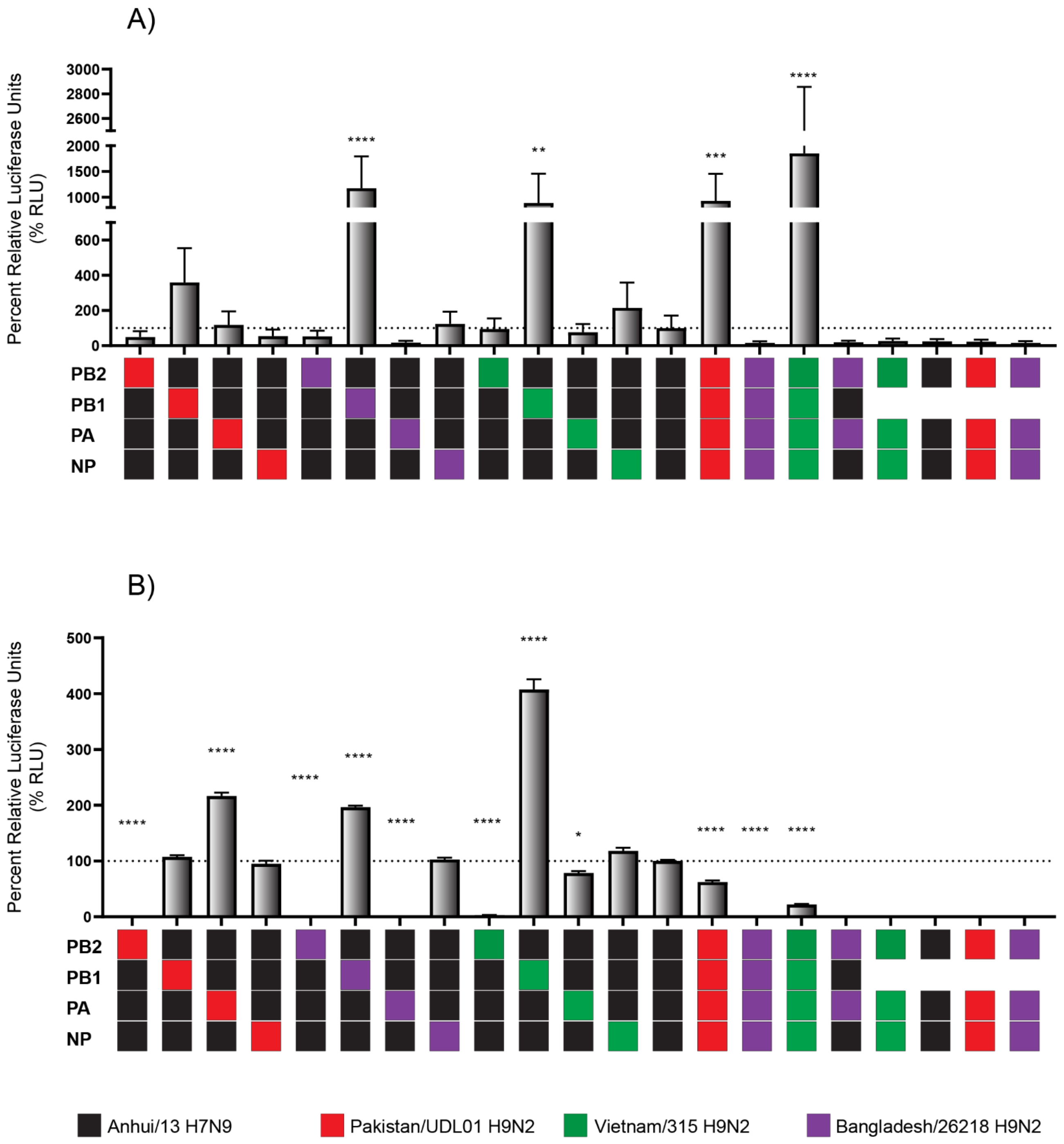

Supplementary Figure 7. Minireplicon assays of the ribonucleoprotein (RNP) complexes of H7N9 Anhui/13 and H9N2 UDL/08, H9N2 Vietnam/315, H9N2 Bangladesh/26218 and their additional RNP combinations. The RNP complexes were reconstituted by transfecting chicken DF-1 (A) and human HEK-293T (B) cells and incubating at 39°C and 37°C, respectively. Luciferase activities were measured 24 hrs post-transfection. RNP complexes without PB1 served as negative control. The polymerase activity of Anhui/13 H7N9 was set at 100% and percent relative luciferase units (% RLU) was calculated. The data shown are a representative of two independent experiments. Ordinary One-way ANOVA was carried out by comparison with Anhui/13 H7N9. * denotes P-value <0.05; ** denotes P value <0.005; *** denotes P-value <0.001; **** denotes P-value <0.0001.

Supplementary materials

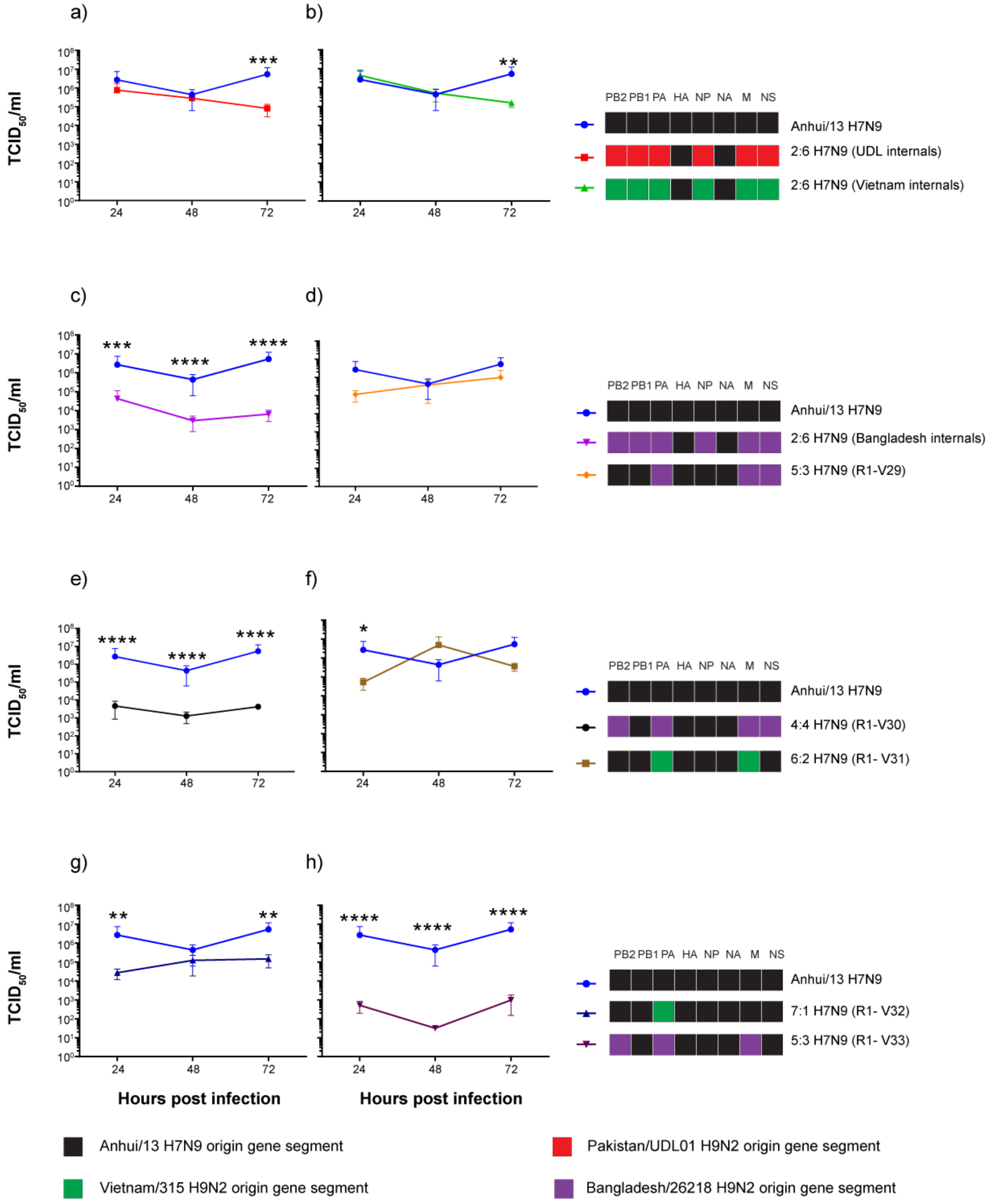

Supplementary Figure 8. Replication analysis of Anhui/13 H7N9, 2:6 H7N9 viruses and the reassortant viruses in human Calu-3 cells. Virus replication of 2:6 H7N9 viruses containing the HA and NA from H7N9 and the remaining internal gene cassettes from either Pakistan, Vietnam or Bangladesh (A-C) H9N2 viruses or novel reassortant viruses recovered by plaque purification from C1 chicken swabs (D-H), see Table 1. Cells were infected at a MOI of 0.01 with each virus and cell supernatant was harvested at 24, 48 and 72hr post-infection. Viral titres in the cell supernatant were determined by TCID50/ml. Each time point corresponds to the mean of four biological replicates with standard deviations indicated. Virus titres of reassortant viruses compared to parental Anhui/13 H7N9 virus is shown in all panels A-H. The genotype of each reassortant virus is shown as a combination of black, red, green, and violet colours. Two-way ANOVA with multiple analysis was performed comparing every group to Anhui; * indicates P-value = 0.0237; ** indicates P-value < 0.005, **** indicates P-value <0.0001.

Supplementary Table 1. Primer and probes to detect segment-specific viral genes from the individual H9N2 viruses and Anhui/13 (H7N9).

Virus	Target gene	Primer/probe	Sequence (5' - 3')to detect
A/chicken/ Bangladesh/26218/2014	M	F	GACCCAAACAACATGGACAAG
		R	CAGTTGAGTAACTGAGTGCAAC
		Bang-P	HEX - CAGTCAAG+CTATA+CAA+AAA+GTT+AAAGA - BHQ1
		Anhui-P	FAM - CGGTTAAA+TTATA+CAA+GAA+ACT+GAAGA - BHQ1
	NP	F	TTCGAAAGAGCAACCATTATGG
		R	TGGTCTGGCACTTTCCATC
		Bang-P	HEX - A+AAGGGAATACCGAGGGCAGGAC+AT – BHQ1
		Anhui-P	FAM - A+CAGGAAATACTGAGGGTAGAAC+GT – BHQ1
	NS	F	ATTGAAAGCAAATTTCAGTGTGA
		R	TCAGTATGTCCTGGAAGAGAAGG
		Bang-P	HEX - TTGATCGACT+GGA+CACCTTAATACT – BHQ1
		Anhui-P	FAM - TCAATCGGCT+TGA+AGCCCTGATACT – BHQ1
	PA	F	TGGACTTTGAGGATTGYAAAGATG
		R	CCTTGTTGAAYTCAYTCTGGATC
		Bang-P	HEX - TTAGTGA+TCT+AAA+GCA+ATAT+GACAGT – BHQ1
		Anhui-P	FAM - TCAACGA+CTT+GAA+ACA+GTAC+AACAGT – BHQ1
	PB1	F	GAGGGAATACARGCAGGAG
		R	AAAAGCTTGTGAAYTCRAATGT
		Bang-P	HEX - TG+AACAG+ATTCTATAGGAC+TTGCAAGCT – BHQ1
		Anhui-P	FAM - TA+GATAG+GTTCTATAGAAC+CTGCAAATT – BHQ1
	PB2	F	CAGCAGGAGTGGAATCTGC
		R	CTCCTTGCCCTATCAACACATTA
		Bang-P	HEX - AGAGGATT+CTTAATTCTAGGGAA+GGA – BHQ1
		Anhui-P	FAM - AGGGGATTTCTGATTCTGGGCAA+AGA – BHQ1
A/Chicken/ Vietnam/315/2015	M	F	GGATGGGAGTGCAACTGC
		R	GACGATCAAGAATCCACAATATCAAG
		Viet-P	HEX - AAGTGA+TCCTCT+TGT+TGTTGCA – BHQ1
		Anhui-P	FAM - AAGTGA+GCCTCT+AGT+CGTTGCA – BHQ1
	NP	F	AGCGTTCAACCCACTTTC
		R	TCTACCCTCAGTATTTCCTGTAAA
		Viet-P	HEX - TCGGTACAGCGAAA+TCTCCCCTTCGA – BHQ1
		Anhui-P	FAM - TCAGTACAGAGAAA+CCTTCCTTTCGA – BHQ1
	NS	F	CAGCACTCTTGGTCTGGA
		R	AATGCTTCATCTGACTCTTCC
		Viet-P	HEX - TAAGAGCTGCCACTCGTGAAGGGA – BHQ1
		Anhui-P	FAM - TCAGAACTGCCACGCGTGAAGGAA – BHQ1
	PA	F	GAGAAGGTGGACTTTGAGGA
		R	TCTGGATCCAACATGCTAGTG
		Viet-P	FAM - AAGATGTCAACGAC+TTGAAACA+GTACAA – BHQ1
		Anhui-P	HEX - AGGATGTCAACGAC+CTGAAACA+ATACGA – BHQ1
	PB1	F	GCACCGAATCATGAGGGAATA
		R	TTCGAATGTTCCTGTCCGATTTA
		Viet-P	HEX - CAGGAGT+GGA+CAGGTT+TTATAGAAC – BHQ1
		Anhui-P	FAM - CAGGAGT+AGA+TAG+GTT+CTATAGAAC – BHQ1
	PB2	F	GTAGCTGGAGGGACAAGC
		R	TCCCGGTGTGTACATTTGC
		Viet-P	HEX - TATAT+CGA+AGT+ATT+ACATTT+GACCCA – BHQ1
		Anhui-P	FAM - TATATCGA+GGT+GTT+GCATTTGACCCA – BHQ1
A/chichi/Pakistan/UDL-1/08	All	All	Published: Bhat et al 2021 (34)

Blue bold text indicates nucleotide differences compared to Anhui-13; + indicates Locked Nucleic Acids (LNA) to the right

Supplementary Table 2: Amino acid differences in the 8 internal 'core' proteins between Anhui and the 2:6 viruses

Protein	aa Position	A/Anhui/1/13	A/chicken/Vietnam/315/15	A/chicken/Pakistan/UDL-01/08	A/Env/Bangladesh/26218/15
PB2	81	T			I
	105	T		K	
	113	K		Q	Q
	147	I		V	V
	167	V		I	
	175	R		K	K
	191	K	E	E	E
	195	D		E	
	271	T	I		
	288	Q			L
	292	V		I	
	293	R			G
	318	R			K
	340	R	K		
	389	K		R	R
	440	K		R	R
	451	I			V
	457	V			I
	559	N	T	T	T
	570	M		I	I
	575	M		I	
	588	A	V		
	598	V		T	T
	627	K	E	E	E
	648	V		L	L
	660	K		R	
	676	M		A	A
	707	A			P
PB1	12	V			I
	14	V		A	A
	20	T		I	I
	48	K		Q	Q
	54	K		R	I
	113	I		V	
	149	V		I	
	156	T			A
	158	N		S	S
	171	M	V		
	172	D		E	E
	191	V			I
	194	N		S	
	215	R		K	K
	257	A		T	T

	261	S		G	A
	363	K		R	R
	368	V		I	I
	383	K		E	E
	384	S		A	P
	386	R			K
	387	E		K	K
	397	I	M		
	430	K		R	R
	525	V	I	I	I
	552	I		V	
	573	A		S	S
	584	R			H
	621	Q		R	R
	628	M		L	L
	638	D		E	E
	655	M			I
PA	3	D		N	N
	37	S		A	A
	61	T		I	I
	63	I		V	V
	86	M		L	L
	100	A	V	V	
	160	D		E	E
	208	T			A
	213	R		K	K
	262	R	K	K	K
	263	T			A
	269	R	K		K
	272	N		D	D
	319	E		D	D
	323	I		V	V
	327	E		K	K
	337	T		A	S
	356	R		K	K
	367	K			R
	379	V			M
	388	N		S	S
	391	K		R	
	394	N	D	G	D
	400	P		S	
	405	C		S	S
	409	N		S	S
	423	V		I	I
	430	E	D		
	475	A		T	
	550	L			I

	554	V		I	I
	561	M		L	
	607	L		M	M
	610	E			D
	626	K			E
	631	G		S	S
	684	E		G	G
NP	34	S		G	G
	52	N		Q	Q
	109	I			V
	136	L		I	I
	183	V		I	I
	186	I		V	V
	210	E		D	D
	214	R			K
	217	I	V		
	239	M	V		
	251	A		V	
	257	I			L
	316	I			V
	352	M		V	V
	353	V		I	I
	371	M		V	V
	372	E		D	D
	373	A			T
	377	N		S	S
	406	V	I	I	I
	417	N		S	S
	430	T		K	K
	433	T			A
	482	N	S	S	S
	494	E			D
M1	37	A		V	
	46	I	L	L	L
	54	P		S	S
	59	I		V	V
	98	K		R	
	139	T		N	N
	140	A		T	T
	142	G		V	V
	167	T		A	A
	192	M		V	V
	219	V		I	I
	224	N		S	S
	227	T	A	A	A
	242	N		K	K
	246	V		L	L

	248	L		M	M
M2	10	P	L	L	L
	12	R	K		
	13	T		N	N
	16	E		G	V
	18	N		R	K
	20	S		K	
	21	G		D	D
	24	E	D	D	D
	28	V		I	F
	31	N		S	
	32	I			V
	65	T		K	
	68	M		V	
	82	N	I	S	
	88	D			A
	97	K		E	E
NEP	3	S		P	
	14	T	M	M	M
	22	R	G	G	G
	36	E			K
	48	A		T	
	49	A	T	V	V
	76	I	L		
	81	E			G
	3	S		P	
NS1	27	M	L	L	L
	47	S		G	
	55	R		E	S
	56	T	A		
	59	R		C	
	60	E		A	A
	62	K		R	R
	63	H		Q	Q
	67	R			Q
	71	E		G	
	72	E		K	
	76	A			T
	77	F		L	L
	80	S	N	T	T
	84	V	M		
	87	P		S	
	91	T		S	S
	103	L		F	F
	106	I		M	M
	111	I	V	V	V
	112	T			A

	119	M			I
	124	V		M	M
	127	N	T	T	T
	129	T		I	I
	139	N		D	D
	142	E		D	D
	143	A		T	T
	145	I		V	
	155	A			S
	163	L	I		
	171	D	N	Y	
	172	K	E	E	E
	179	E	G	G	G
	180	I		V	V
	185	F	L	L	L
	193	R			Q
	205	S		N	
	206	S	N		
	207	D	Y	N	
	211	R		G	G
	212	S	P	P	P
	215	S		P	P
	216	T	P	P	P
	217	K	E		
	218	*	S	Q	Q
	219	-	K	K	K
	220	-	R	W	W
	221	-	E	K	K
	222	-	M	M	M
	223	-	E	A	A
	224	-	R	R	R
	225	-	T	T	T
	226	-	V	I	I
	227	-	K	K	K
	228	-	P	S	S
	229	-	E	E	E
	230	-	V	I	V
	231	-	R	*	*
	232	-	R		
	233	-	T		
	234	-	K		
	235	-	M		
	236	-	A		
	237	-	N		
	238	-	*		
